# Co-Culture of Plant Beneficial Microbes as Source of Bioactive Metabolites

**DOI:** 10.1038/s41598-017-14569-5

**Published:** 2017-10-30

**Authors:** F. Vinale, R. Nicoletti, F. Borrelli, A. Mangoni, O. A. Parisi, R. Marra, N. Lombardi, F. Lacatena, L. Grauso, S. Finizio, M. Lorito, S. L. Woo

**Affiliations:** 1 Istituto per la Protezione Sostenibile delle Piante (IPSP-CNR), Portici, Italy; 2 Consiglio per la Ricerca in Agricoltura e l’Analisi dell’Economia Agraria, Rome, Italy; 3 Dipartimento di Farmacia, Università degli Studi di Napoli Federico II, Napoli, Italy; 4 Dipartimento di Agraria, Università degli Studi di Napoli Federico II, Portici, Italy; 5 Stazione Zoologica “Anton Dohrn”, Napoli, Italy

## Abstract

In microbial cultures the production of secondary metabolites is affected by experimental conditions, and the discovery of novel compounds is often prevented by the re-isolation of known metabolites. To limit this, it is possible to cultivate microorganisms by simulating naturally occurring interactions, where microbes co-exist in complex communities. In this work, co-culturing experiments of the biocontrol agent *Trichoderma harzianum* M10 and the endophyte *Talaromyces pinophilus* F36CF have been performed to elicit the expression of genes which are not transcribed in standard laboratory assays. Metabolomic analysis revealed that the co-culture induced the accumulation of siderophores for both fungi, while production of M10 harzianic and iso-harzianic acids was not affected by F36CF. Conversely, metabolites of the latter strain, 3-*O*-methylfunicone and herquline B, were less abundant when M10 was present. A novel compound, hereby named harziaphilic acid, was isolated from fungal co-cultures, and fully characterized. Moreover, harzianic and harziaphilic acids did not affect viability of colorectal cancer and healthy colonic epithelial cells, but selectively reduced cancer cell proliferation. Our results demonstrated that the co-cultivation of plant beneficial fungi may represent an effective strategy to modulate the production of bioactive metabolites and possibly identify novel compounds.

## Introduction

Filamentous fungi are able to synthetize a wide range of chemically different metabolites and some of these compounds can be applied as anticancer or antibiotic substances^1,2^. However, there is a high rate of redundancy that results in the frequent re-isolation of already known compounds^3^. Apparently, only some fungal biosynthetic genes are transcribed in laboratory conditions, while other remain silent and are not expressed *in vitro* ^3,4^. Genome sequencing has now revealed that prolific antibiotic producers, such as fungi and actinomycetes, have several gene clusters coding for secondary metabolites that are not generally expressed, but can be activated using specific elicitors^4^.

To overcome these limitations, it is possible to cultivate microorganisms by simulating naturally occurring conditions, where microbes co-exist within complex communities, generally referred as the “microbiome”^3–7^.

Bioactive secondary metabolites play a crucial role during the interactions within such microbial communities, and may be related to different mechanisms, like competition for space/nutrients, parasitism and antagonism, as well as the induction of plant defence responses^5^.

Many useful drugs have been obtained from natural sources, particularly plants and microbes. Fungi belonging to the genus *Trichoderma* are among the most studied and applied beneficial microbes used in agriculture as biopesticides and biofertilizers^8–10^. *Trichoderma* spp. can improve plant growth and resistance to pathogens, enhance nutrient assimilation and abiotic stress tolerance^8,11^. The efficacy of beneficial *Trichoderma* strains has been often related to the great array of secondary metabolites that they are able to produce, belonging to diverse classes of chemical compounds^12–14^ and showing different biological activities^12–17^.

Beneficial microbes include also fungal endophytes that colonize plants, determining numerous advantages for the host, including protection against pathogens, growth promotion, induction of disease resistance, and production of bioactive compounds with antibiotic and antitumor properties^18,19^. Also known as a fungal endophyte, *Talaromyces pinophilus* is able to produce a variety of bioactive metabolites, including alkaloids, peptides, lactones, polyketides and miscellaneous structure type compounds, with different chemical and biological activities^20^.

Plant endophytes and symbionts represent promising sources of novel bioactive compounds, and recent studies have revealed that many important drugs, first considered to be produced by the plants, are possibly derivatives of the interaction between plants and microbes, or between microbes^18–23^.

In this work, the co-cultivation of two plant beneficial fungi (*T. harzianum* M10 and *T. pinophilus* F36CF) was used to stimulate the synthesis of novel secondary metabolites not normally accumulated in standard single cultures grown in laboratory conditions. Moreover, the use of metabolomic analysis allowed the rapid identification of novel compounds produced during fungal-fungal interaction, resulting in a time-saving screening of fungal metabolites whose biosynthesis was modulated by co-cultivation. A novel tetramic acid not previously detected in either M10 or F36CF culture filtrates, was isolated and its structure plus absolute configuration were determined by spectroscopic methods. Finally, the anti-cancer activity of the isolated metabolites was also examined.

## Results and Discussion

**Figure 1:**
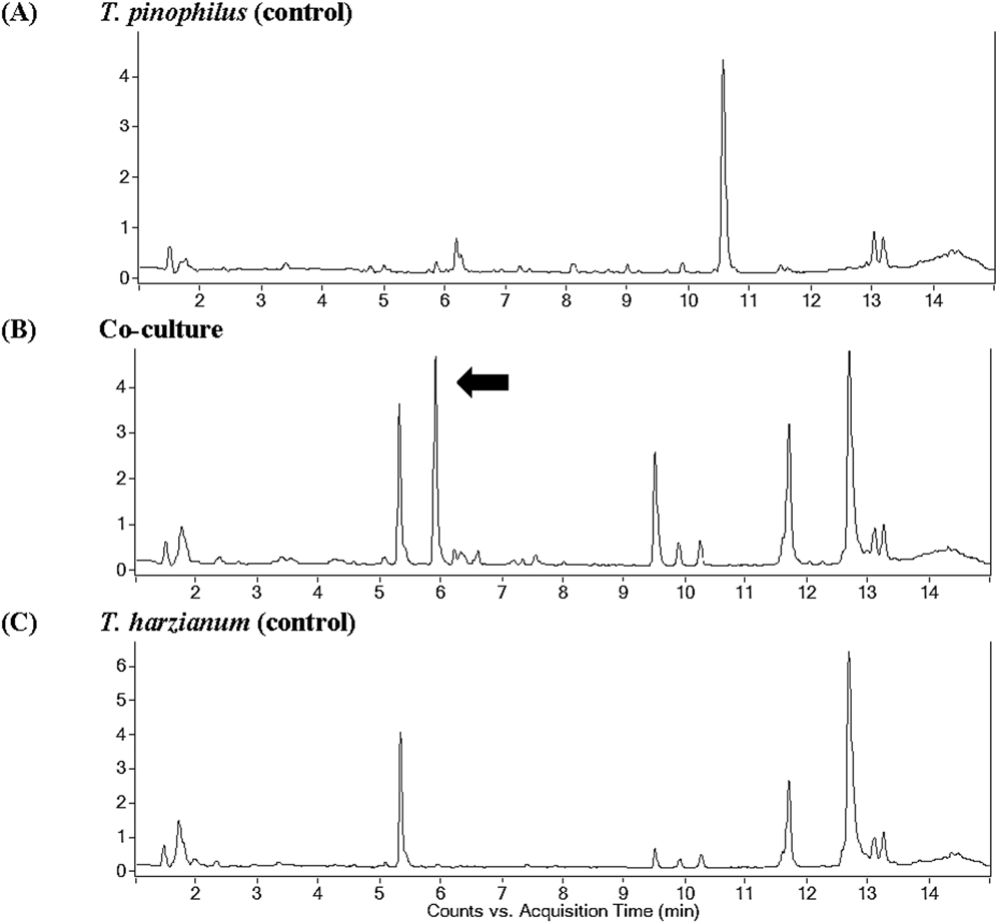
Total Ion Chromatogram (TIC) in positive ion mode (*m/z* 100–1700 uma) of the culture filtrate of: (**A**) *T. pinophilus* (top); (**B**) co-culture (middle); (**C**) *T. harzianum* (bottom). The black arrow indicates the new peak observed only in the co-culture.

In the current study, a metabolomic approach was used to analyze the effects of growing single or combined cultures of *T. harzianum* M10 and *T. pinophilus* F36CF on the production of fungal secondary metabolites in liquid culture. The performed principal component analysis (PCA) clustered the samples in three groups, representing the two single cultures (*Trichoderma* or *Talaromyces*) and the co-culture (*Trichoderma* and *Talaromyces*) (data not shown). The chromatograms of the two single fungal cultures (controls) and the co-culture are reported in Fig. 1, where significant differences in terms of peak intensity were observed in the time of retention ranging at 5–7 and 9–13 min. Interestingly, an extra peak was present only in the co-culture chromatogram at 5.9 min (indicated by the black arrow in Fig. 1).

Untargeted analysis revealed that the presence of the other fungus modified the metabolome of the counterparty. This finding supports the hypothesis that microbial interactions modulate the biosynthesis of secondary metabolites.

**Figure 2:**
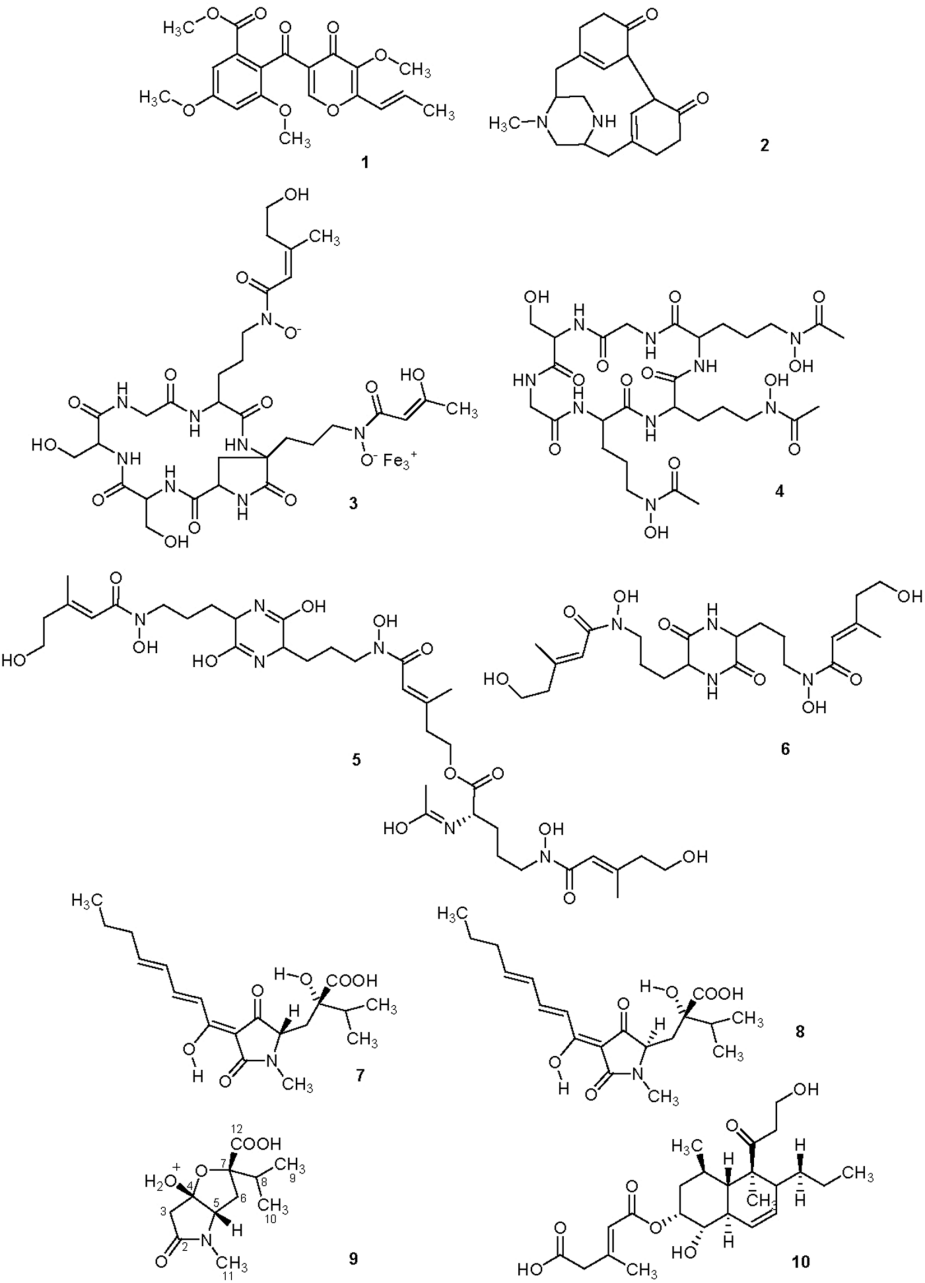
Chemical structures of 3-O-methylfunicone (**1**), herquline B (**2**), ferrirubin (**3**), ferricrocin (**4**), coprogen B (**5**), dimerumic acid (**6**), harzianic acid (**7**), iso-harzianic acid (**8**), the new metabolite named harziaphilic acid (**9**) and trichoharzin (**10**).

Table 1 reports the secondary metabolites discriminating single and combined treatments. Metabolites (Fig. 2; 1–10) were identified by LC-MS qTOF analysis (operating in positive ion mode), by comparing the data with known compounds present in an in-house database including over 4000 fungal secondary metabolites. Identification of fungal metabolites was confirmed by using the MassHunter Qualitative Analysis Software and selecting matching having a score ≥ 95%.

**Table 1:** Secondary metabolites identified in the *T. pinophilus*/*T. harzianum* co-culture by LC-MS qTOF analysis. Identifications were confirmed by comparing results with known compounds present in an in-house database including over 4000 fungal secondary metabolites and selecting matching with a score ≥ 95%.

	Compound	Molecular formula	Exact Mass	*m/z*	RT (min)	Production in co-culture*
**1**	3-O-Methylfunicone	C_20_H_20_O_8_	388.1158	389.1231 [M+H]^+^	10.5	**↓**
				411.1049 [M+Na]^+^		
				427.0786 [M+K]^+^		
				799.2205 [2 M+Na]^+^		
**2**	Herquline B	C_19_H_26_N_2_O_2_	314.1994	315.2066 [M+H]^+^	8.2	**↓**
**3**	Ferrirubin	C_41_H_67_N_9_O_17_	957.4655	958.4718 [M+H]^+^	5.9	**↑**
				980.4529 [M+Na]^+^		
**4**	Ferricrocin	C_28_H_47_N_9_O_13_	717.3293	718.3365 [M+H]^+^	5.1	**↑**
				740.3178 [M+Na]^+^		
**5**	Coprogen B	C_33_H_54_N_6_O_12_	726.3804	727.3881 [M+H]^+^	5.3	**↑**
				749.3683 [M+Na]^+^		
				1453.7672 [2 M+H]^+^		
**6**	Dimerumic acid	C_22_H_36_N_4_O_8_	484.2531	485.2604 [M+H]^+^	5.4	**↑**
				507.2414 [M+Na]^+^		
**7**	Harzianic acid	C_19_H_27_NO_6_	365.1839	366.1914 [M+H]^+^	12.7	**↔**
				388.1731 [M+Na]^+^		
				404.1475 [M+K]^+^		
				753.3575 [2 M+Na]^+^		
**8**	Iso-harzianic acid	C_19_H_27_NO_6_	365.1839	366.1912 [M+H]^+^	11.5	**↔**
				388.1726 [M+Na]^+^		
				404.1467 [M+K]^+^		
				753.3563 [2 M+Na]^+^		
**9**	New metabolite (harziaphilic acid)	C_11_H_17_NO_5_	243.1108	244.1188 [M+H]^+^	5.9	**ON**
				266.0998 [M+Na]^+^		
				487.2289 [2 M+H]^+^		
				509.2102 [2 M+Na]^+^		
**10**	Trichoharzin	C_25_H_38_O_7_	450.2617	451.2693 [M+H]^+^	9.5	**↑**
				473.2507 [M+Na]^+^		
				489.2243 [M+K]^+^		
				923.5133 [2 M+Na]^+^		

*^↑^Increased production of the metabolite in co-culture vs. single culture.

^**↓**^Decreased production of the metabolite in co-culture vs. single culture. ^↔^Unchanged production of the metabolite in co-culture vs. single culture.

ON: metabolite produced *ex novo* in co-culture.

The secondary metabolites identified in this study and released during the interaction of the two microbes belonged to different classes of natural products, including pyrones, tetramic acids and nitrogen containing molecules. The biosynthesis occurring during the co-culturing condition varied in relation to: (i) the specific compound or class of molecules produced; (ii) the presence of another microbe that elicited the production; (iii) the viability of the elicitor (part of the fungal tissue or released products); and (iv) the balance between elicited biosynthesis (or degradation) and biotransformation rates^24^.

The major metabolites produced by *T. pinophilus* F36CF in single culture were 3-*O*-methylfunicone (OMF) and herquline B. Both compounds were down regulated by the presence of *T. harzianum* M10 (Table 1). OMF is a well-known γ-pyrone derivative previously isolated from *Talaromyces* spp.^25,26^, and it was the most abundant secondary metabolite biosynthesized by F36CF in single culture (Fig. 1, peak at 10.5 min). OMF exhibited notable antibiotic and antitumor properties^27,28^, and recently an insecticidal effect has also been observed^26^, thus expanding the range of its biological activities. Herquline B, earlier purified from a strain of *Penicillium herquei*, is a nitrogen-containing metabolite acting as platelet anti-aggregation factor^29^. The reduced amounts of both OMF and herquline B in the co-cultures are probably due to degradation by *Trichoderma,* a well known producer of several degrading enzymes^30^.

Ferrirubin is a ferrichrome-type siderophore that was up regulated by F36CF during the interaction with M10. This molecule is known to be produced by many filamentous fungi and its main function has been related to iron transportation^31^.

The siderophores ferricrocin, coprogen B and dimerumic acid were all up-regulated in *T. harzianum* M10 when co-cultivated with *T. pinophilus* F36CF. The production of these natural compounds by beneficial microbes is important during the interaction with the plant, as they aid in iron solubilization with a consequent plant growth promotion effect^11^. Moreover, the production of microbial siderophores can also suppress the growth of other microorganisms through iron competition^16^, a function possibly explaining the up-regulation of these bioactive compounds.

The *Trichoderma* metabolite harzianic acid (HA) was also identified in both single and combined cultures (Table 1). Although this metabolite exhibited both iron-chelating and plant growth promotion activities^32,33^, its production was not affected by the presence of the competing fungus. A similar result was also obtained with its stereoisomer, named iso-harzianic acid (iso-HA; Table 1), known to induce disease resistance in plants^34^.

Among the *Trichoderma* metabolites induced by the presence of F36CF, significant homology was found with trichoharzin, a polyketide first purified from a *T. harzianum* strain isolated from the marine sponge *Mycale cecilia* ^35^. This compound showed an experimental high resolution mass at *m/z* 473.2507 [M+Na]^+^, corresponding to the molecular formula of C_25_H_38_NaO_7_. Trichoharzin exhibited modest cytotoxic activity against different cancer cell lines and its biosynthesis was affected by media salinity or stress conditions^36,37^. Similarly, the induction of a trichoharzin homologue in our study could be related to the occurrence of a stress condition possibly due to the presence of a microbial competitor.

Interestingly, the chromatogram of the co-culture filtrate indicated the presence of one additional peak corresponding to a compound that was absent in each of the controls (Fig. 1, peak indicated by black arrow). This new tetramic acid represents a metabolite not yet isolated from natural sources or chemically synthesised and induced by the interaction of *T. harzianum* M10 with *T. pinophilus* F36CF, was isolated and fully characterized. The co-culture filtrate was concentrated and fractionated by semipreparative HPLC. Each fraction was monitored by LC-MS qTOF to detect the presence of the new compound with an experimental high resolution mass at *m/z* 244.1180 [M+H]^+^. Fraction 4 was further purified by HPLC to obtain 9 mg of the pure metabolite named herein harziaphilic acid (**9**; Table 1, Fig. 2), that represents the main discriminating compound in the M10-F36CF co-culture.

The molecular formula of **9** (4.5 mg/L) was generated according to the algorithm of the Mass Hunter Qualitative software from the high-resolution ESI-MS spectrum as C_11_H_17_NO_5_. The proton NMR spectrum of **9** (DMSO-*d*_6_) showed signals for 15 protons: a methyl singlet at δ 2.66, suggesting an *N*-methyl group; two methyl doublets (δ 0.82 and 0.75) and a 1H septet (δ 2.03), which were part of an isopropyl group as shown by the COSY spectrum; an isolated AB system (δ 2.49 and 2.42, *J* = 17.1 Hz); and the signals of a CH-CH_2_ fragment (δ 3.79, dd, *J* = 7.5 and 3.1 Hz; δ 2.44, dd, *J* = 14.0 and 7.5 Hz; and δ 1.81, dd, *J* = 14.0 and 3.1 Hz). These data also suggested the presence of two exchangeable protons in **9**. The ^13^C NMR spectrum showed the presence of two carbonyl groups (δ 176.4 and 170.1) as the sole *sp* ^2^ carbon atoms in the molecule. This, combined with the 4 unsaturations implied by the molecular formula, indicated a bicyclic structure for **9**.

Connection of the structural units identified so far was provided by the HMBC experiment (Table 2 and Figure S2a). The presence of the pyrrolidone ring was demonstrated by the coupling of the *N-*methyl protons at δ 2.66 (H_3_-11) with the CO carbon atom at δ 170.1 (C-2) and the methine carbon atom at δ 69.8 (C-5), and by the coupling of the proton at δ 2.49 (H-3a) with the same carbon atom C-2 and C-5, and with a non-protonated carbon atom resonating at δ 107.1 (C-4). The chemical shift of this latter carbon was suggestive of a ketal carbon. The coupling of the methyl protons H_3_-9 and H_3_-10 with the oxygen-bearing proton at δ 91.2 (C-7) showed that the isopropyl group was linked to C-7; the coupling of isopropyl methine proton H-8 with the carboxy carbon atom at δ 176.4 (C-12) extended the chain to this atom. Finally, the HMBC correlations of the methylene proton at δ 1.81 (H-6b) with C-4, C-5, C-7, C-8 and C-12 completed the carbon skeleton of the molecule.

**Table 2:** ^1^H and ^13^C NMR spectral data of harziaphilic acid (9).

Position	**9** (DMSO-*d*_6_). 50 °C			**9-** *d*_4_ (CD_3_OD)		
	δ_C_ (mult.)	δ_H_ (J in Hz)	HMBC^a^	ROESY	δ_C_ (mult.)	δ_H_ (J in Hz)
2	170.1 (C)	—	—	—	173.6 (C)	—
3	43.5 (CH_2_)	2.49 (d, 17.1)	2, 4, 5	—	44.3b	—
		2.42 (d, 17.1)	2, 4	—		
4	107.1 (C)	—	—	—	108.7 (C)	—
5	69.8 (CH)	3.79 (dd, 7.5, 3.1)	2, 4, 7	6a, 6b, 11	71.6 (CH)	3.98 (dd, 7.6, 3.1)
6	34.4 (CH_2_)	2.44 (dd, 14.0, 7.5)	12	5, 6b		2.68 (dd, 14.5, 7.6)
		1.81 (dd, 14.0, 3.1)	4, 5, 7, 8, 12	5, 6a, 8, 9, 10, 11	35.1 (CH2)	2.05 (dd, 14.5, 3.1)
7	91.2 (C)	—	—	—	93.2 (C)	—
8	33.2 (CH)	2.03 (septet, 6.8)	7, 9, 10, 12	6b, 9, 10	35.1 (CH)	2.12 (septet, 6.9)
9	17.5 (CH_3_)	0.82 (d, 6.8)	7, 8, 10	6b, 8	17.8 (CH3)	0.91 (d, 6.9)
10	16.6 (CH_3_)	0.75 (d, 6.8)	7, 8, 9	6b, 8	17.0 (CH3)	0.85 (d, 6.9)
11	26.7 (CH_3_)	2.66 (s)	2, 5	5, 6b	27.7 (CH3)	2.81 (s)
12	176.4 (C)	—	—	—	179.5 (C)	—

Abbreviations: s, singlet; d, doublet; dd, doublet of doublets.

^a^HMBC correlations, optimized for 8.3 Hz, are from proton stated to the indicated carbon.

^b^δ_C_ determined from the HMBC experiment.

Since the molecular formula of **9** required two rings and five oxygen atoms, the structure was completed with an oxygen bridge between C-4 and C-7 to form a five-membered cyclic hemiketal, and a free carboxyl group at C-12. The alternative possibility of an oxygen bridge between C-4 and C-12 to form the six-membered cyclic hemiketal ester (**9a** in Figure S2b) was ruled out because it did not fit the coupling constants of H-5 with H-6a and H-6b (the large coupling constant between the cis protons H-5 and H-6a and the small coupling constant between the trans protons H-5 and H-6b are not compatible with a six-membered ring). Structure **9** was further supported by LC-MS/MS analysis (Figure S10). All the prominent fragment ions observed could be rationalized as originating from the loss from **9** of one or more molecules of water, ketene (C-2 and C-3), and formic acid (C-12); in particular, the loss of formic acid confirms the presence of a free carboxylic acid function.

**Figure 3:**
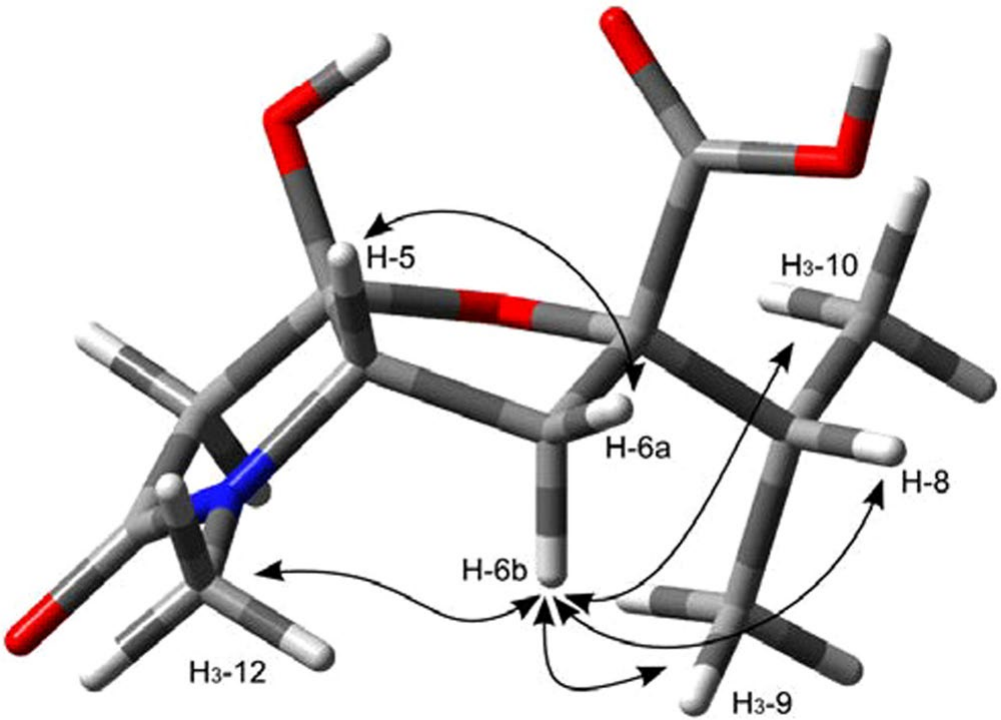
The minimum energy conformer of compound **9** as determined by quantum mechanical calculations. Arrows represent the most significant correlations detected in the NOESY spectrum.

**Figure 4:**
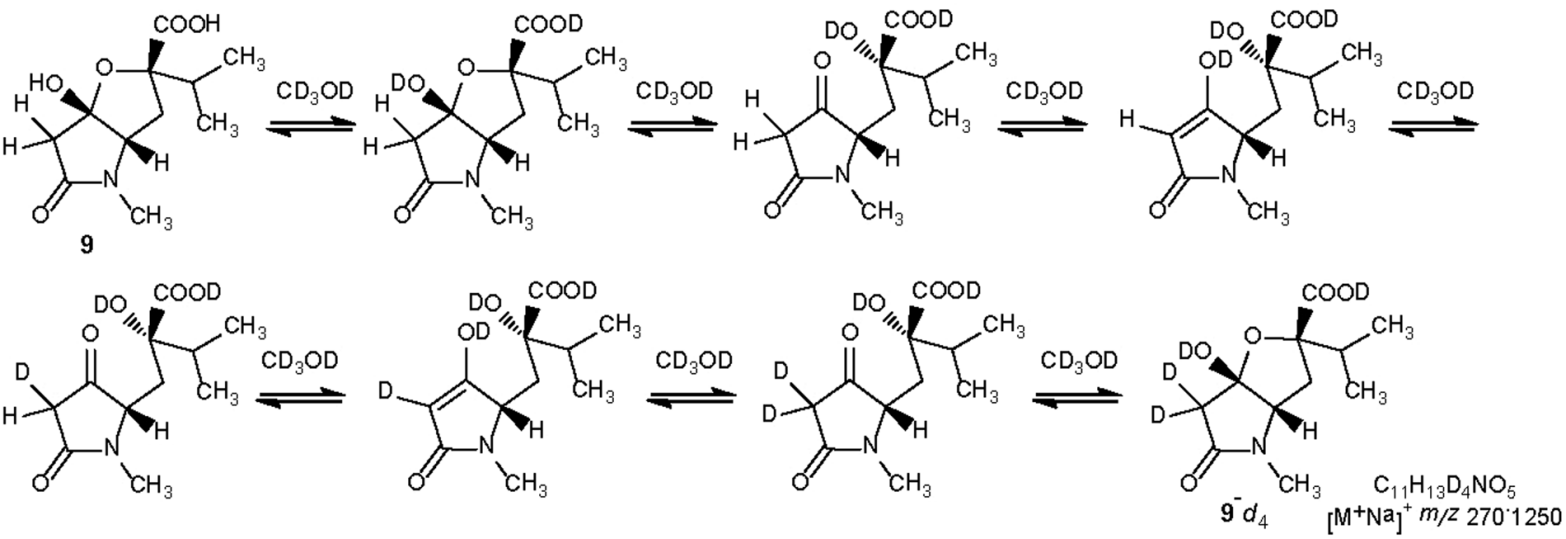
Hemiacetals equilibrium of harziaphilic acid (**9**) and deuterium exchanges obtained after 3 days in CD_3_OD.

The ring junction in **9** was assigned as cis because a trans junction between two five membered rings is highly strained^38^, and therefore strongly disfavored in a molecule in which equilibration between cis- and trans-junction is possible via the open keto form (see also Fig. 4). Determination of relative configuration at C-7 required preliminary stereospecific assignment of protons at C-6, which was possible on the basis of the strong NOESY correlation peak between H-6a and H-5, illustrating their cis relationship (a correlation peak is present between H6b and H-5 as well, but it is by far weaker), and the NOESY correlation peak between H-6a and the *N*-methyl protons H_3_-12 (Fig. 3). The NOESY correlation peaks of H-6b with H-8, H_3_-9, and H_3_-10 then demonstrated that the isopropyl group is cis to H-6b, and therefore trans to H-5.

The absolute configuration of compound **9** was established by quantum-mechanical (QM) prediction of optical rotation. It is becoming clear that in many instances the experimental optical rotation of a molecule can be predicted using time-dependent density functional theory (TDDFT)^39^. However, a reliable prediction requires a detailed examination of the conformational behavior of the molecule under study and a sufficiently high level of theory (e.g. CAM-B3LYP/6-311+(d,p)). Therefore, the conformational space of compound **9** (the 4 *R*,5 *R*,7 *R* enantiomer was randomly chosen for calculations) was preliminary sampled using molecular dynamics (MD). A 10 ps MD simulation in the CVFF force field identified 21 conformers. The geometry of the conformers was then optimized by QM using the program Gaussian 09, Rev. E.01^40^ at the CAM-B3LYP/6-311+(d,p) level. Optimization caused four pairs of conformers to converge to the same structure, reducing the number of different conformers to 17. Optical rotation of each conformer was then calculated by TDDFT using Gaussian 09 at the same theory level. Finally, the predicted optical rotation of **9** was obtained as the Boltzmann-weighted mean of the optical rotations of the individual conformers (Table S1). The predicted optical rotation of (4*R*, 5*R*, 7*R*)- **9** was [α]_D calc_ = −99, compared with an experimental optical rotation of [α]_D exp_ = +57. The comparable magnitude, but opposite sign of the calculated and experimental optical rotations clearly shows that the enantiomer chosen for calculations was not the correct one, and the absolute configuration of compound **9** is therefore (4*S*, 5*S*, 7*S*). It is to be noted that the configuration at C-5 and C-7 of compound **9** matches that at the corresponding carbons of harzianic acid (**7**), suggesting a biogenetic relationship between the two compounds.

An interesting feature of **9**, possibly related to its biological significance, is connected with its capacity of exchanging the two protons at C-3 (besides the two OH protons) with the solvent. This was noticed when CD_3_OD was used as solvent for NMR experiments, and signals for protons at C-3 were not present in the proton NMR spectrum. In addition, the signal of C-3 was not detectable in the ^13^C NMR spectrum recorded in CD_3_OD (coupling with deuterium generates a multiplet which is submerged by noise), but its chemical shift could be determined indirectly from the HMBC spectrum (Table 2). A further experimental proof of the incorporation of 4 deuterium atoms exchange is given by the mass spectrum obtained infusing a CD_3_OD solution of **9** in the ESI source. A peak at *m*/*z* 270.1250 was observed in the MS spectrum, corresponding to the mass of the sodium adduct of **9**-*d*_4_ (C_11_H_13_D_4_NO_5_Na) (Figure S3).

The hypothesized mechanism for the exchange is depicted in Fig. 4. The hemiketal function of compound **9** may open to give a 2,4-pyrrolidinedione, an 1,3-dicarbonyl compound that can easily exchange protons with deuterium via its enol form (the hydroxyl and carboxyl protons are also exchanged). Restoring the hemiketal function gives the tetradeuterated harziaphilic acid **9**-*d*_4_.

Harziaphilic acid is a new simple tetramic acid derivative synthetised in presence of F36CF elicitors by *T. harzianum* M10 that produces also other tetramic acids (harzianic and iso-harzianic acids^32–34^).

Secondary metabolites containing the tetramic acid core scaffold (2,4-pyrrolidine-2,4- dione) have been isolated from various microbial sources. Tetramic acid derivatives have a wide distribution and demonstrate different biological properties, including antibacterial, antifungal, antiviral and antitumoral activities. In some cases this class of molecules are responsible for the pigmentation of given moulds and sponges^41^. For these important features these molecules play a significant role in ecological interactions and have attracted considerable attention from biologists and chemists^42,43^.

Interestingly, a strain of *T. harzianum* grown in co-culture with calli of *Catharathus roseus* produced an unexpected antimicrobial tetramic acid, called trichosetin^44^. This compound showed phytotoxic activity on various plant species and represents a N-desmethyl analogue of equisetin, a tetramic acid isolated from *Fusarium equiseti* ^45,46^. Recently, Whitt *et al*.^47^ isolated three new decalin-type tetramic acid analogues related to equisetin from the co-culture of *F. pallidoroseum* with the bacterium *Saccharopolyspora erythraea*. The three metabolites named N-demethylophiosetin, pallidorosetin A and B were not active against the Gram-positive bacteria *Staphylococcus erythraea* and *S. aureus* and did not exhibit cytotoxicity against the NCI-60 cell line^47^.

**Figure 5:**
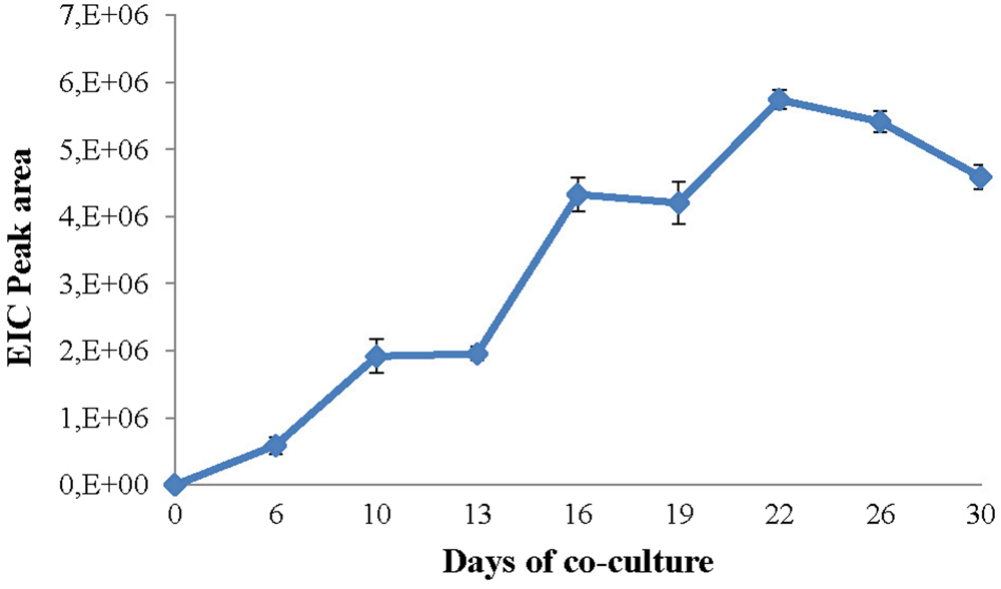
Production of harziaphilic acid during the M10–TP1 co-culture, from 0 to 30 days. The amount of harziaphilic acid is expressed as peak area of the corresponding compound. Bars indicate standard deviation.

Figure 5 reports the production of harziaphilic acid during the M10 – F36CF co-culture. The amount of harziaphilic acid reached the maximum after 22 days of co-cultivation, then started to decrease. Similar results were obtained also for HA, whereas iso-HA reached the maximum production after 30 days^34^.

**Figure 6:**
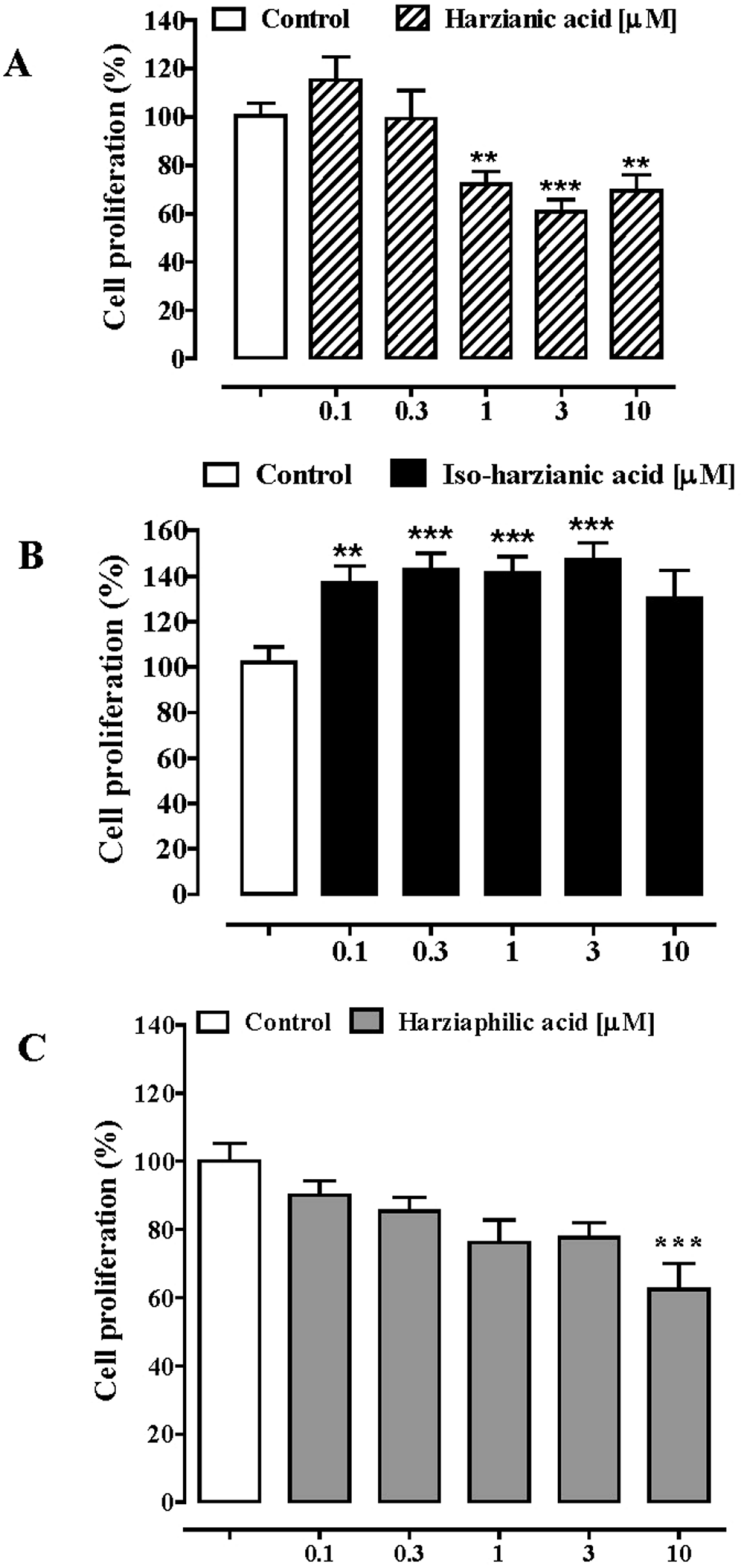
Effect of harzianic acid (0.1–10 μM, 24-h exposure, (**A**) iso-harzianic acid (0.1–10 μM, 24-h exposure, (**B**) and harziaphilic acid (0.1–10 μM, 24-h exposure, (**C**) on Caco-2 cells proliferation. Proliferation rate (expressed as percentage) was studied using the ^3^H-thymidine incorporation assay. Each bar represents the mean ± standard errors mean of three independent experiments. **p < 0.01 and ***p < 0.001 *vs*. control.

**Figure 7:**
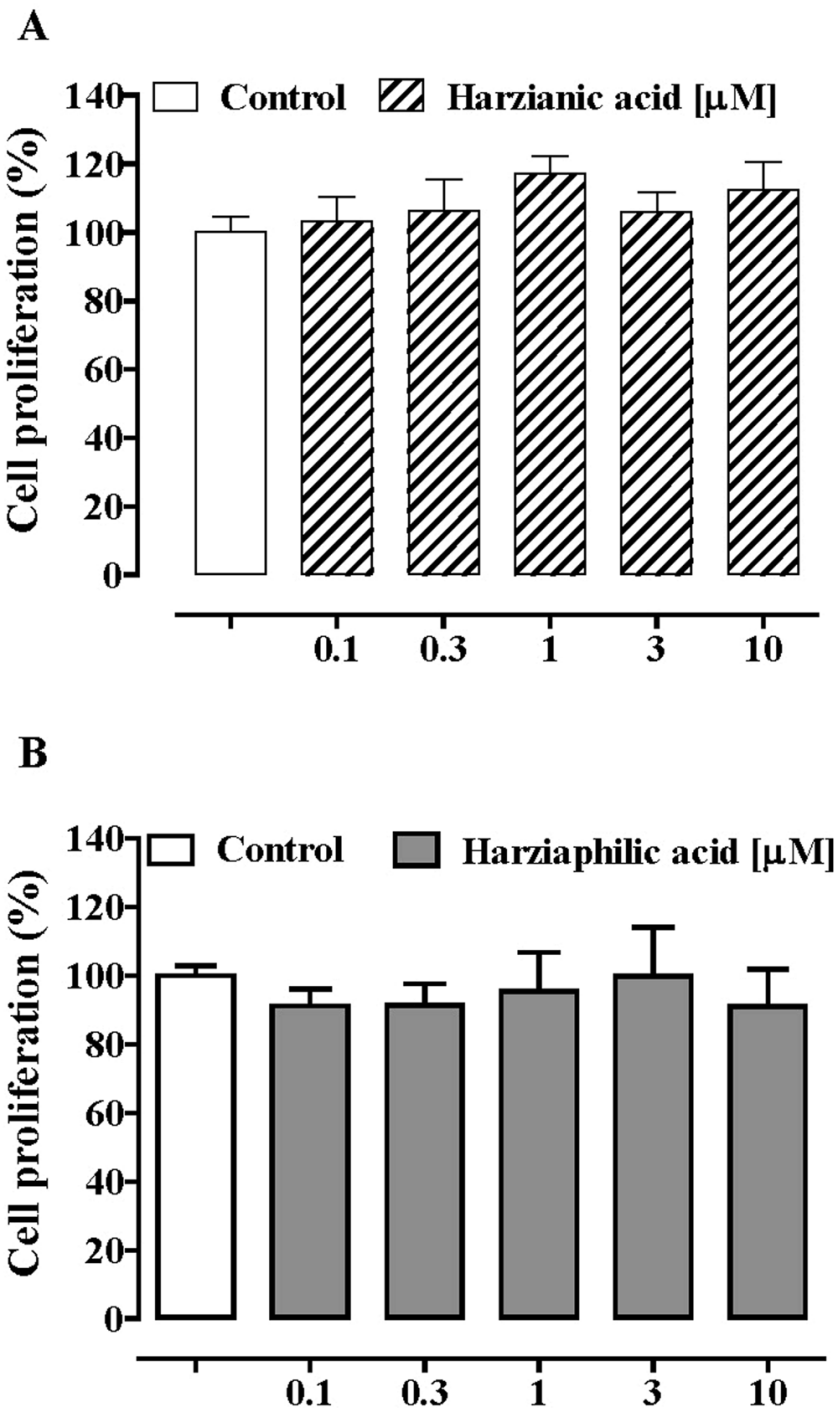
Effect of harzianic acid (0.1–10 μM, 24-h exposure, (**A**) and harziaphilic acid (0.1–10 μM, 24-h exposure, (**B**) on cells proliferation performed on healthy human colonic epithelial cells (HCEC). Proliferation rate (expressed as percentage) was studied using the ^3^H-thymidine incorporation assay. Each bar represents the mean ± standard errors mean of three independent experiments.

Several studies have demonstrated the antiproliferative properties of OMF against a number of human tumor cell lines^25^. Since, to date, no studies have been performed on a possible antineoplastic effect of HA, iso-HA and harziaphilic acid, we hereby have evaluated the effect of HA, iso-HA and harziaphilic acid on colorectal cancer (Caco-2) cell viability and proliferation. HA, iso-HA and harziaphilic acid, at the concentration range of 0.1–10 µM, did not affect the Caco-2 cell viability (Figure S10). HA and harziaphilic acid (0.1–10 µM), in a concentration dependent manner, significantly reduced Caco-2 cell proliferation (Fig. 6). Conversely, iso-HA (0.1–10 µM), at all concentrations, increased Caco-2 cell proliferation. To investigate the selectivity of HA and harziaphilic acid effects on tumoral versus nontumoral cells, the same concentrations of HA and harziaphilic acid (0.1–10 µM) were tested on healthy human colonic epithelial cells (HCEC). HA and harziaphilic acid did not significantly affect HCEC viability (Figure S11) and proliferation (Fig. 7).

Nonaka *et al*.^48^ demonstrated an enhanced accumulation of different fungal compounds with co-cultures of *T. harzianum* and *Penicillium pinophilum* (=*T. pinophilus*), underlining the importance of this method to both obtain novel natural products as well as increase the accumulation of known compounds. Similarly, co-cultures of plant beneficial microbes, such as fungal endophytes and *Trichoderma* spp., may be used to modulate and/or stimulate the production of bioactive compounds *ex novo*. Moreover, metabolomic analysis can be effectively used to study the changes in the metabolic profile, and guide the selection of active fractions. This ecologically driven approach may help to discover novel bioactive metabolites promote the production of the chemical diversity in microbes, as well as to find elicitors of specific or desired biosynthetic pathways.

In conclusion, in the present study, the metabolome of two beneficial fungi grown together has been analysed in order to simulate naturally occurring conditions in which two (or more) microbes co-exist within a complex community. Our results indicated that the presence of the fungal counterpart may significantly influence metabolite production, i.e. by inducing siderophores accumulation. A novel natural compound, named harziaphilic acid, specifically induced by the fungal interaction was isolated and fully characterized. Moreover, evidence supporting a possible selective anti-proliferative effect of HA and harziaphilic acid, but not iso-HA, was demonstrated on colorectal carcinoma cells. This is the first report clearly showing this important biological activity of such secondary metabolites.

## Material and Methods

### Fungal strains and culturing conditions

*T. harzianum* strain M10 was isolated from composted hardwood bark suppressive to *Pythium irregulare*. Sequence analysis of the internal transcribed spacers (ITS) of the rDNA indicated 99% similarity with GenBank sequences of *T. harzianum* confirming the identity of this species^33^. *T. pinophilus* F36CF was recovered, according to a standard protocol for isolation of endophytic fungi, from a secondary branch of a strawberry tree (*Arbutus unedo)* collected in the isle of Favignana, Sicily. In particular, some segments of the plant tissue were surface sterilized by dipping in a 1% solution of sodium hypochlorite and washed extensively with sterile distilled water, then placed onto potato dextrose agar (PDA, HI-MEDIA Mumbai, India) plates amended with 1% lactic acid. The plates were incubated at 25 °C and fungal plugs were transferred into fresh plates until pure endophyte culture was obtained. The fungus was identified according to morphological characters and molecular analyses using primers for rDNA-ITS and β-tubulin gene sequences^26^.

The beneficial fungi were maintained on PDA at room temperature and sub-cultured bimonthly.

Liquid cultures were prepared in 500 mL-Erlenmayer flasks containing 100 mL of potato-dextrose broth (PDB, HIMEDIA) and inoculated with a mycelial plug (7 mm^2^) from a fresh PDA culture of both fungal strains. After 21 days of incubation at 25 °C in static condition, the cultures were filtered at 0.45 µm and the filtrate was analyzed by LC-MS qTOF. In parallel, monocultures of M10 and F36CF were grown using the same conditions. Five replicates of single or combined cultures were prepared for the metabolomic analysis. For harziaphilic acid detection a separate experiment has been performed using 100 mL of PDB in five replicates of single or combined cultures. Every 3 days (starting on day 6 – conclusion after 21 days of incubation) a 0.5 mL-sample was aseptically collected, filtered (0.45 μm, Millipore) and stored at −20 °C until use. The co-culture experiments were monitored measuring the biomass production in terms of dry weight of filtered mycelia (data not shown).

 A scale-up of the co-culture was carried out using 5,000 L-Erlenmayer flask containing 1,000 L of PDB. After 21 days of growth, the cultures were filtered and concentrated using a freeze drier until a reduction to 1/10 of the starting volume was achieved.

### Metabolites extraction and isolation

The concentrated culture filtrate was exhaustively extracted with ethyl acetate (EtOAc). The exhaust and the crude organic extract (dried with Na_2_SO_4_ and concentrated under reduced pressure at 37 °C) were analysed to isolate the unidentified compound with [M+H]^+^ at *m/z* 244.1180, specifically induced in the co-culture. The new metabolite was found in the water exhaust, that contained the more polar components not removed by partitioning with EtOAc. The water exhaust was further separated by HPLC (Agilent HP 1260 with a UV-VIS detector at 215 nm; Agilent Technologies, Torrance, CA, USA), using a C18 column (Phenomenex Prodigy, 10 µm, 250 × 10 mm) at a flow rate of 3 mL/min with a mobile phase gradient of 20% acetonitrile/ 80% water (ACN/H_2_O) for 12 min, 20% to 90% ACN over 16 min. Both solvents were acidified with 0.1% formic acid. The unidentified metabolite was present as major compound in fraction 4 (85% of purity) that was further purified by HPLC using a C18 column (Agilent Poroshell, 2.7 µm, 100 × 2.1 mm) at a flow rate of 1 mL/min and the same mobile phase gradient described above, to obtain pure harziaphilic acid (**9**; 4.5 mg/L). All the solvents used were from Sigma-Aldrich (Germany) unless otherwise stated.

### LC-MS/MS Q-TOF analysis

All analyses were conducted on an Agilent HP 1260 Infinity Series liquid chromatograph equipped with a DAD system (Agilent Technologies, Santa Clara, CA, USA) coupled to a Q-TOF mass spectrometer model G6540B (Agilent Technologies) with a Dual ESI source. Separations were performed on a Zorbax Eclips Plus C18 column, 4.6 × 100 mm, with 3.5 μm particles (Agilent Technologies). The analyses were done at a constant temperature of 37 °C and using a linear gradient system composed of A: 0.1% (v/v) formic acid in water, and B: 0.1% (v/v) formic acid in acetonitrile. The flow was 0.6 mL min^−1^, 95% A graduating to 100% B in 12 min, 100% B 12–15 min, 95% A 15–17 min and equilibrating 95% A 17–20 min. The UV spectra were collected by DAD every 0.4 s from 190 to 750 nm with a resolution of 2 nm. The MS system was equipped with a Dual Electrospray Ionization (ESI) source and operated with Agilent MassHunter Data Acquisition Software, rev. B.05.01 in the positive mode. Mass spectra were recorded in the range *m/z* 100–1600 as centroid spectra, with 3 scans per second. Two reference mass compounds were used to perform the real-time lock mass correction, purine (C_5_H_4_N_4_ at *m/z* 121.050873, 10 μmol L^−1^) and hexakis (1 H,1 H, 3H-tetrafluoropentoxy)-phosphazene (C_18_H_18_O_6_N_3_P_3_F_24_ at *m/z* 922.009798, 2 μmol L^−1^). The capillary was maintained at 4000 V, fragmentor voltage at 180 V, cone 1 (skimmer 1) at 45 V, Oct RFV at 750 V. Gas temperature was 350 °C during the run at 11 L min^−1^, and the nebulizer was set at 45 psig. The injected sample volume was 5 μL.

MS/MS spectra were simultaneously recorded for confirmation purposes of compound **9**, using the operating parameters described above, unless otherwise stated. The instrument was operated in the range *m/z* 100–1000, recording 2 spectra per second in targeted acquisition mode (targeted mass: 244.1197, Z = 1, RT 5.88 ± 0.5 min). The sample collision energy was set at 20 V.

### Data analysis

LC-MS data were evaluated using MassHunter Qualitative Analysis Software B.06.00 and compared to known compounds included in an in-house database. The database contains information of about 4000 known secondary metabolites isolated from more than 80 different fungal genera, and recorded according to their name, molecular formula, monoisotopic mass and producing organism. Positive identifications of fungal metabolites were reported if the compound was detected with a mass error below 10 ppm and with a sufficient score.

### NMR and Optical rotations

NMR spectra were determined on a Varian Unity Inova spectrometers at 700 and 500 MHz; chemical shifts were referenced to the residual solvent signal (CD3OD: δH 3.31, δC 49.00; DMSO-d6: δH 2.54; δC 40.45). For an accurate measurement of the coupling constants, the one-dimensional 1 H NMR spectra were transformed at 64-K points (digital resolution: 0.09 Hz). Homonuclear ^1^H connectivities were determined by a COSY and z-TOCSY10 experiments. Through-space ^1^H connectivities were evidenced using a ROESY experiment with a mixing time of 450 ms. The HSQC spectra were optimized fo 1JCH = 142 Hz, and the HMBC experiments for 2,3JCH = 8.3 Hz.

Optical rotations were measured at 589 nm on a Perkin–Elmer 192 polarimeter using a 10-cm microcell.

### Quantum mechanical calculation of optical rotation

The conformational space of (4 *R*,5 *R*,7 *R*)-**9**, the enantiomer arbitrarily chosen for calculations, was sampled using molecular dynamics (MD). A 10 ns MD simulation at 300 K was carried out in the CVFF force field using the Insight II/Discover package (BIOVIA, San Diego, CA 92121, USA). The coordinates were saved every 50 ps, giving 200 structures. Each structure saved from MD was then minimized. The calculation generated 21 different conformers, differing in the puckering of the two five-membered ring and in the network of hydrogen bonds. Density functional theory (DFT) calculations were performed using the program Gaussian 09^40^. The geometry of each conformers from MD was optimized using the CAM-B3LYP functional, the 6–311G+(d,p) basis set, and the IEF-PCM model for the solvent, MeOH. The results of vibrational frequency analysis confirmed that all conformers were in a true energy minimum, and were used to obtain their free energies. Four pairs of conformers converged to the same structure during quantum-mechanical optimization, reducing the total number of conformers to 17. The Cartesian coordinates of each conformer are reported in Table S2. Optical rotation of each DFT-optimized conformer was then calculated using time-domain density functional theory (TDDFT) at the same theory level. The optical rotation calculated for each conformer, along with their internal energy and free energy are reported in Table S1. Finally, the theoretical optical rotation was determined as the weighted mean of individual optical rotations, calculated using Boltzmann statistics (T = 298 K) on the basis of their DFT relative free energies.

### Cell cultures

A human colon adenocarcinoma cell line (Caco-2, ATCC from LGC Standards, Milan, Italy) and a healthy human colonic epithelial cell line (HCEC, from Fondazione Callerio Onlus, Trieste, Italy) were used. The cells were routinely maintained at 37 °C in a 5% CO_2_ atmosphere in 75 cm^2^ polystyrene flasks in Dulbecco’s modified Eagle’s medium (DMEM). For Caco-2 cells, DMEM was supplemented with 10% fetal bovine serum (FBS), 100 U/mL penicillin and 100 μg/mL streptomycin, 1% non-essential amino acids, and 2 mM L-glutamine. For HCEC, DMEM was supplemented with 10% FBS, 100 U/mL penicillin, 100 μg/mL streptomycin, 20 mM Hepes [4-(2-hydroxyethyl)-1-piperazineethanesulphonic acid], 2 mM L-glutamine and 1 mM Na pyruvate. The media were changed every 48 h in conformity with the manufacturer’s protocols.

### Cell viability assay (MTT assay)

Cell viability was evaluated by measuring the mitochondrial reductase activity (MTT assay). Briefly, Caco-2 and HCEC were seeded in presence of 10% FBS in 96-well plates at a density of 1 × 10^4^ cells per well and allowed to adhere for 48 h. After, cells were incubated with increasing concentrations of HA, iso-HA or harziaphilic acid (0.1–10 μM), for 24 hours and subsequently with MTT (250 μg/mL, for 1 h at 37 °C). After solubilization in DMSO, the mitochondrial reduction of MTT to formazan was quantitated at 490 nm (iMarkTM microplate reader, BioRad, Italy). All results are expressed as percentage of cell viability (n = 3 experiments including 8–10 replicates for each treatment).

### Cell proliferation assay

Cell proliferation was evaluated in Caco-2 and HCEC using ^3^H-thymidine incorporation assay as previously described^49^. Briefly, cells were seeded in 24-well plates at a density of 1 × 10^4^ cells/well (Caco-2) and 2 × 10^4^ cells/well (HCEC) in DMEM supplemented with 10% FBS and grown for 48 h. Successively, cells were washed three times with 200 μL of phosphate buffered saline (PBS) and then 200 μL of serum-free DMEM was added to each well. After 24 h of serum starvation, the cells were washed three times with PBS and incubated with DMEM supplemented with 10% FBS containing HA, iso-HA or harziaphilic acid (0.1–10 μM) in presence of ^3^H-thymidine (1 µCi/well) for 24 h. Cells were scraped in 2 M NaOH and collected in plastic miniature vials (PerkinElmer) filled up with liquid for scintillation counting (UltimaGold^®^, PerkinElmer). Results are expressed as percentage of cell proliferation. The treatments were carried out in triplicate and three independent experiments were performed.

### Statistical analysis

Results are expressed as mean ± S.E.M of n experiments. For analysis of multiple treatment means, one-way analysis of variance (ANOVA) followed by Tukey–Kramer multiple comparison tests was used (using GraphPad Prism Software; Inc. San Diego, CA, USA). A p-value less than 0.05 was considered significant.

## Electronic supplementary material

Supplementary Information
