# Supplementary material for: Co-Culture of Plant Beneficial Microbes as Source of Bioactive Metabolites
Source: Sci Rep. 2017 Oct 30;7:14330. doi: 10.1038/s41598-017-14569-5 (PMC5662714; doi:10.1038/s41598-017-14569-5)
Supplement: Supplementary file 1 — Supplementary Information [file 41598_2017_14569_MOESM1_ESM.docx]

**Supplementary data**

**CO-CULTURE OF PLANT BENEFICIAL MICROBES AS SOURCE OF BIOACTIVE METABOLITES**

**F. Vinale^1,4*^, R. Nicoletti^2^, F. Borrelli^3^, A. Mangoni^3^, O.A. Parisi^3^, R. Marra^4^, N. Lombardi^1^, F. Lacatena^4^, L. Grauso^5^, S. Finizio^3^, M. Lorito^1,4^, S.L. Woo^1,3^**

*^1^Istituto per la Protezione Sostenibile delle Piante (IPSP-CNR), Portici (NA), Italy*

*^2^Consiglio per la Ricerca in Agricoltura e l’Analisi dell’Economia Agraria, Rome, Italy*

*^3^Dipartimento di Farmacia, Università degli Studi di Napoli Federico II, Napoli, Italy*

*^4^Dipartimento di Agraria, Università degli Studi di Napoli Federico II, Portici (NA), Italy*

*^5^Stazione Zoologica "Anton Dohrn" , Villa Comunale, Napoli , Italy.*

* Corresponding author: Francesco Vinale

Phone number: +39 081 2539338

Fax number: +39 081 2539339

e-mail address: francesco.vinale@ipsp.cnr.it

Via Università 100 – 80055 Portici (Naples) – Italy

**Abstract**

In microbial cultures the production of secondary metabolites is affected by experimental conditions, and the discovery of novel compounds is often prevented by the re-isolation of known metabolites. To limit this, it is possible to cultivate microorganisms by simulating naturally occurring interactions, where microbes co-exist in complex communities. In this work, co-culturing experiments of the biocontrol agent *Trichoderma harzianum* M10 and the endophyte *Talaromyces pinophilus* F36CF have been performed to elicit the expression of genes which are not transcribed in standard laboratory assays. Metabolomic analysis revealed that the co-culture induced the accumulation of siderophores for both fungi, while production of M10 harzianic and iso-harzianic acids was not affected by F36CF. Conversely, metabolites of the latter strain, 3-*O*-methylfunicone and herquline B, were less abundant when M10 was present. A novel compound, named harziaphilic acid, was isolated from fungal co-cultures, and fully characterized. Moreover, harzianic and harziaphilic acids did not affect viability of colorectal cancer and healthy colonic epithelial cells, but selectively reduced cancer cell proliferation. Our results demonstrate that the co-cultivation of plant beneficial fungi may represent an effective strategy to modulate the production of bioactive metabolites and possibly identify novel compounds.

**Figure S1**. MS qTOF spectra of 3-O-methylfunicone (**1**), herquline B (**2**), ferrirubin (**3**), ferricrocin (**4**), coprogen B (**5**), dimerumic acid (**6**), harzianic acid (**7**), iso-harzianic acid (**8**), the new metabolite named Harziaphilic acid (**9**) and trichoharzin (**10**).

**Figure S2.** (a) HMBC correlations detected for compound **9**; (b) the alternative structure of **9** with the oxygen bridge between C-4 and C-12 (**9a**).

**Figure S3.** High-resolution ESI mass spectrum of compound **9** in CD_3_OD solution. The peak at *m*/*z* 270 shows incorporation of four deuterium atoms in **9**. Peaks with three and two deuterium atoms (*m*/*z* 269 and 268, respectively) are due to fast exchange of the hydroxyl and carboxyl deuterium atoms of **9**-*d*_4_ with residual non-deuterated solvents present in the source.

**Figure S4.** ^1^H NMR spectrum of compound **9** (700 MHz, DMSO-*d*_6_, 50 °C).

**Figure S5.** ^13^C NMR spectrum of compound **9** (175 MHz, DMSO-*d*_6_, 50 °C).

**Figure S6.** COSY spectrum of compound **9** (700 MHz, DMSO-*d*_6_, 50 °C).

**Figure S7.** HSQC spectrum of compound **9** (700 MHz, DMSO-*d*_6_, 50 °C).

**Figure S8.** HMBC spectrum of compound **9** (700 MHz, DMSO-*d*_6_, 50 °C).

**Figure S9.** NOESY spectrum of compound **9** (700 MHz, DMSO-*d*_6_, mixing time 400 ms, 50 °C).

**Figure S10**. MS-MS data of harziaphilic acid (**9**).

**Figure S11**. Effects of harzianic acid (0.1–10 μM, 24-h exposure, A), iso-harzianic acid (0.1–10 μM, 24-h exposure, B) and harziaphilic acid (0.1–10 μM, 24-h exposure, C) on Caco-2 cell viability. Cell viability (expressed as percentage) was studied using the MTT assay. Each bar represents the mean± standard errors mean of three independent experiments.

**Figure S12.** Effects of harzianic acid (0.1–10 μM, 24-h exposure, A), and harziaphilic acid (0.1–10 μM, 24-h exposure, B) on healthy human colonic epithelial cells (HCEC) viability. Cell viability (expressed as percentage) was studied using the MTT assay. Each bar represents the mean± standard errors mean of three independent experiments.

**Table S1.** Relative internal energies (Δ*E*), relative free energies (Δ*G*), and optical rotations of the conformers of (4*R*,5*R*,7*R*)-**9** calculated at the CAM-B3LYP/6-311+(d,p) level.

**Table S2.** Cartesian coordinates of the 17 conformers of (4*R*,5*R*,7*R*)-**9** optimized at the CAM-B3LYP/6-311+(d,p) level.

**Figure S1**. MS qTOF spectra of 3-O-methylfunicone (**1**), herquline B (**2**), ferrirubin (**3**), ferricrocin (**4**), coprogen B (**5**), dimerumic acid (**6**), harzianic acid (**7**), iso-harzianic acid (**8**), the new metabolite named harziaphilic acid (**9**) and trichoharzin (**10**).

3-O-Methylfunicone (**1**)

Herquline B (**2**)

Ferrirubin (**3**)

Ferricrocin (**4**)

Coprogen B (**5**)

Dimerumic acid (**6**)

Harzianic acid (**7**)

Iso-harzianic acid (**8**)

New metabolite named Harziaphilic acid (**9**)

**
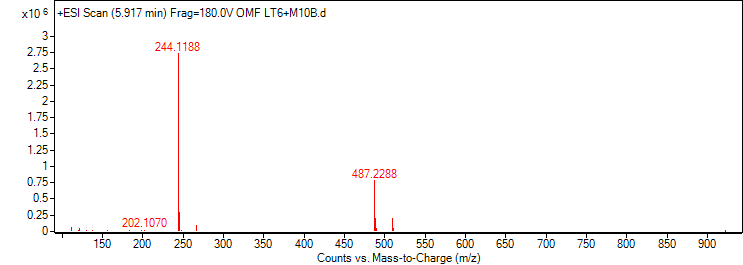
**

Trichoharzin (**10**)

**Figure S2.** (a) HMBC correlations detected for compound **9**; (b) the alternative structure of **9** with the oxygen bridge between C-4 and C-12 (**9a**).

a

b

**Figure S3.** High-resolution ESI mass spectrum of compound **9** in CD_3_OD solution. The peak at *m*/*z* 270 shows incorporation of four deuterium atoms in **9**. Peaks with three and two deuterium atoms (*m*/*z* 269 and 268, respectively) are due to fast exchange of the hydroxyl and carboxyl deuterium atoms of **9**-*d*_4_ with residual non-deuterated solvents present in the ESI source.

**Figure S4.** ^1^H NMR spectrum of compound **9** (700 MHz, DMSO-*d*_6_, 50 °C).





**Figure S5.** ^13^C NMR spectrum of compound **9** (175 MHz, DMSO-*d*_6_, 50 °C).





**Figure S6.** COSY spectrum of compound **9** (700 MHz, DMSO-*d*_6_, 50 °C).





**Figure S7.** HSQC spectrum of compound **9** (700 MHz, DMSO-*d*_6_, 50 °C).


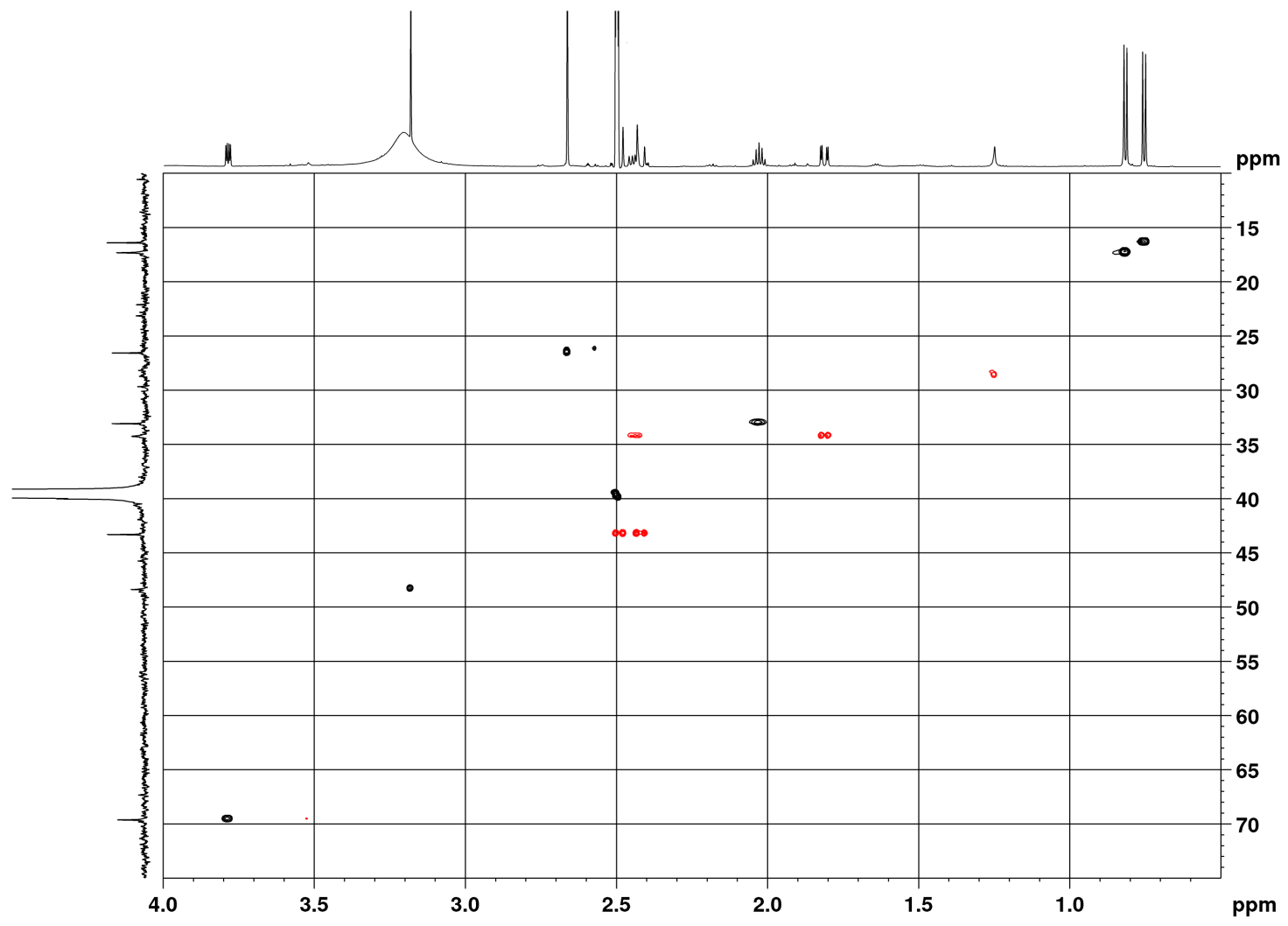


**Figure S8.** HMBC spectrum of compound **9** (700 MHz, DMSO-*d*_6_, 50 °C).





**Figure S9.** NOESY spectrum of compound **9** (700 MHz, DMSO-*d*_6_, mixing time 400 ms, 50 °C).


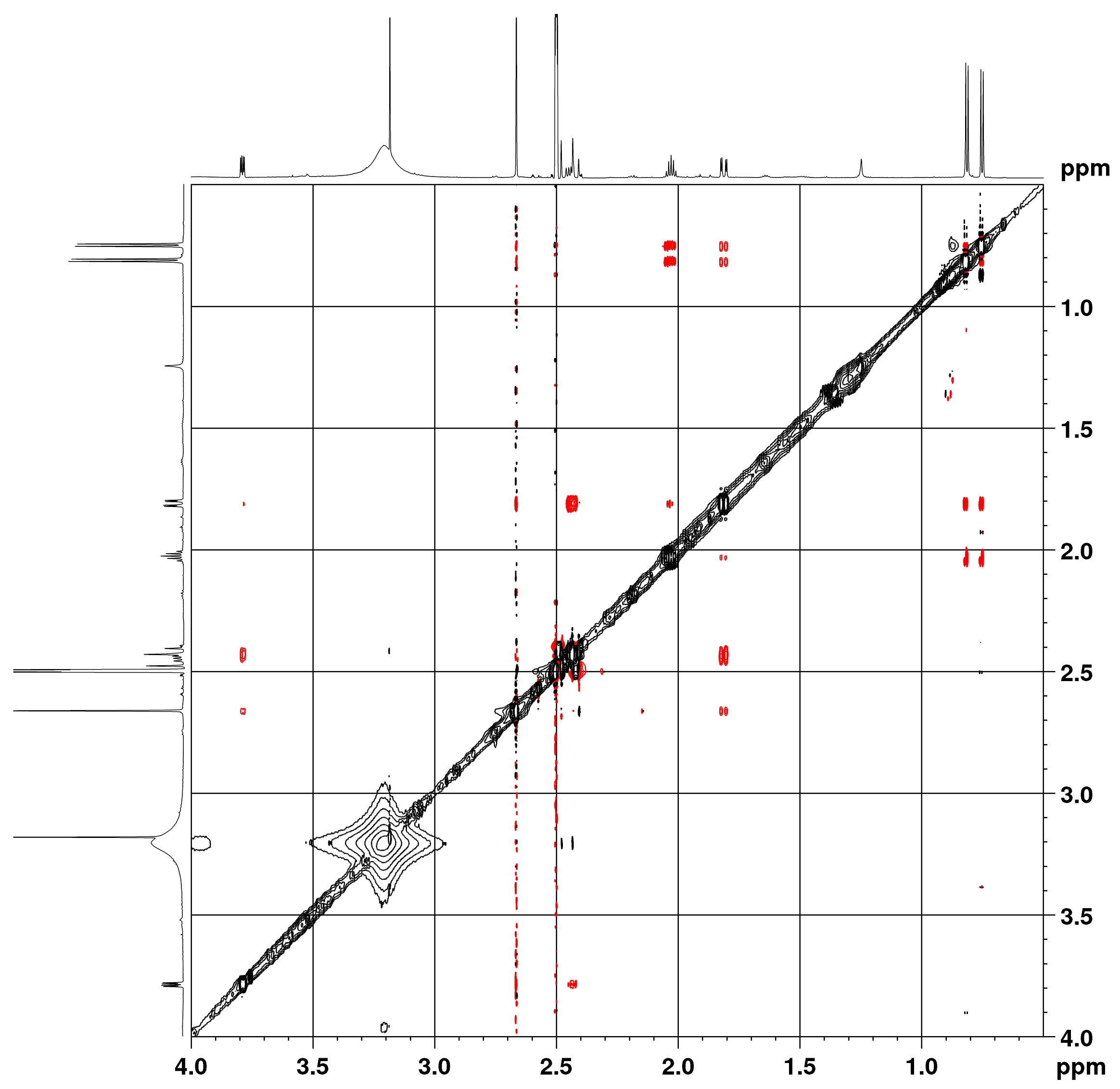


**Figure S10**. MS-MS data of harziaphilic acid (**9**).


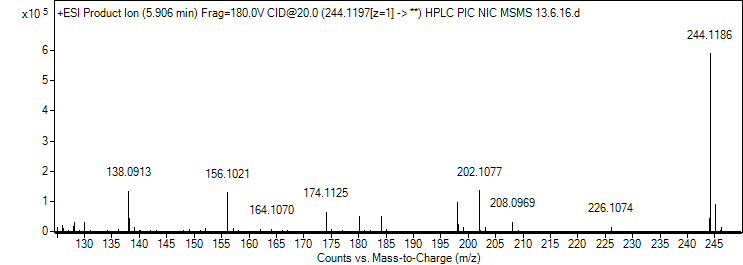


Fragments mass list

244.1186, C_11_H_18_NO_5_ [M + H]^+^ (parent ion)

226.1074, C_11_H_16_NO_4_ [M + H – H_2_O]^+^

208.0969, C_11_H_14_NO_3_ [M + H – 2H_2_O]^+^

202.1077, C_9_H_16_NO_4_ [M + H – C_2_H_2_O]^+^

198.1223, C_10_H_16_NO_3_ [M + H – HCOOH]^+^

184.0969, C_9_H_14_NO_3_ [M + H – C_2_H_2_O – H_2_O]^+^

156.1021, C_8_H_14_NO_2_ [M + H – HCOOH – C_2_H_2_O]^+^

138.0913, C_8_H_12_NO [M + H – HCOOH – C_2_H_2_O – H_2_O]^+^

**Figure S11**. Effects of harzianic acid (0.1–10 μM, 24-h exposure, A), iso-harzianic acid (0.1–10 μM, 24-h exposure, B) and harziophilic acid (0.1–10 μM, 24-h exposure, C) on Caco-2 cell viability. Cell viability (expressed as percentage) was studied using the MTT assay. Each bar represents the mean± standard errors mean of three independent experiments.


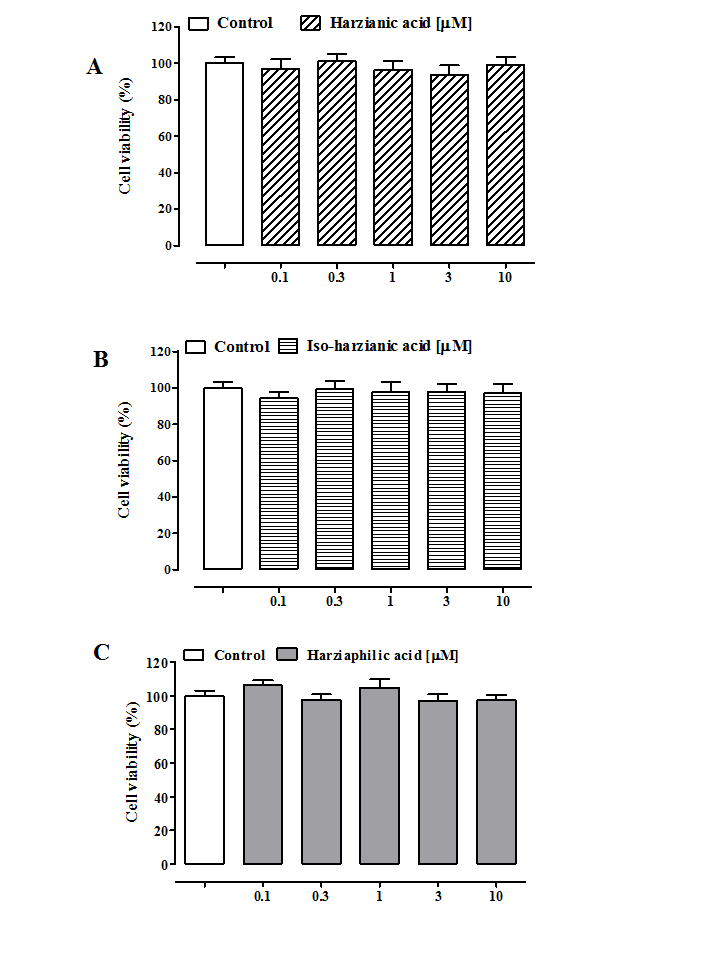


**Figure S12.** Effects of harzianic acid (0.1–10 μM, 24-h exposure, A), and harziaphilic acid (0.1–10 μM, 24-h exposure, B) on healthy human colonic epithelial cells (HCEC) viability. Cell viability (expressed as percentage) was studied using the MTT assay. Each bar represents the mean± standard errors mean of three independent experiments.


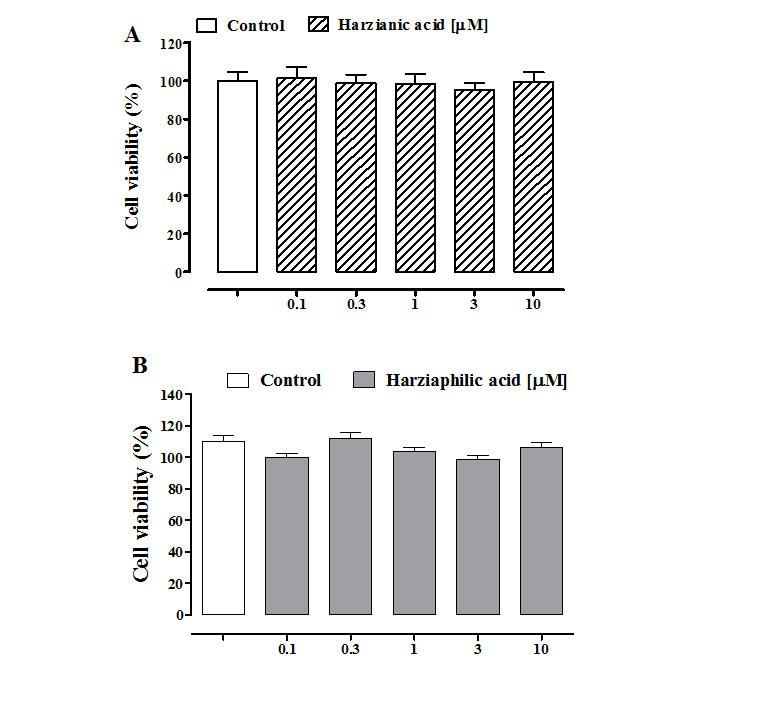


**Table S1.** Relative internal energies (Δ*E*), relative free energies (Δ*G*), and optical rotations of the conformers of (4*R*,5*R*,7*R*)-**9** calculated at the CAM-B3LYP/6-311+(d,p) level.

| Conformer | Δ*E* (kcal/mol) | Δ*G* (kcal/mol) | Boltzmann factor*^a^* | [α] |
| --- | --- | --- | --- | --- |
| 1 | 1.62 | 0.72 | 0.295 | +23.9 |
| 2 | 0.00 | 0.00 | 1.000 | –145.7 |
| 3 | 0.10 | 0.19 | 0.727 | –99.3 |
| 4 | 1.90 | 1.15 | 0.143 | –18.5 |
| 5 | 0.52 | 0.82 | 0.250 | –142.9 |
| 6 | 2.45 | 2.66 | 0.011 | –28.4 |
| 7 | 2.55 | 1.58 | 0.069 | –0.8 |
| 8 | 2.09 | 1.86 | 0.043 | –111.6 |
| 9 | 2.26 | 1.44 | 0.088 | –83.9 |
| 10 | 2.26 | 2.08 | 0.030 | –113.8 |
| 11 | 2.99 | 2.73 | 0.010 | –15.2 |
| 12 | 1.95 | 1.06 | 0.166 | –125.5 |
| 13 | 4.29 | 3.62 | 0.002 | +16.8 |
| 14 | 2.96 | 2.43 | 0.016 | –64.1 |
| 15 | 2.49 | 1.91 | 0.040 | +6.2 |
| 16 | 2.11 | 2.17 | 0.025 | –129.6 |
| 17 | 2.91 | 2.31 | 0.020 | –20.4 |
| **weighted mean** | | | | **–99.0** |
| **experimental value** | | | | **+57.0** |

*^a^* At *T* = 298 K, calculated on ΔG.

**Table S2.** Cartesian coordinates of the 17 conformers of (4*R*,5*R*,7*R*)-**9** optimized at the CAM-B3LYP/6-311+(d,p) level.

Conformation no. 1

C -0.6292639316 1.0146435854 0.6639033841

C -0.3775966364 -0.3522862791 1.3220546547

O 0.201935613 -1.1428372188 0.3083615249

C 1.0288276886 -0.3187392846 -0.5189950078

C 0.3424047054 1.0591441151 -0.5235223172

N -2.0374643472 0.9434991312 0.2983899064

C -2.7092165124 -0.0964824263 0.8452584053

C -1.7409891217 -0.9233086955 1.6673010161

O -3.9046357895 -0.3185490435 0.7185731205

C -2.6917707505 2.0224994834 -0.4094721406

O 0.5069385985 -0.1790125314 2.395656929

C 1.154627919 -1.0146880001 -1.8928087024

C -0.2079125294 -1.2701537423 -2.5342424502

C 2.0706953638 -0.2422887335 -2.8421241299

C 2.4320492809 -0.2254061305 0.1049089678

O 3.0891733629 0.7811235836 0.1821453757

O 2.8821793521 -1.4154508998 0.5095671023

H -0.4612392992 1.8316607253 1.3682486917

H -0.1971845785 1.213691966 -1.4549120463

H 1.0722852075 1.8576144086 -0.420889242

H -1.9713358019 -0.7761258731 2.7238804331

H -1.8256547047 -1.9829534878 1.4313943058

H -2.2127205521 2.178563167 -1.3896804561

H -2.6235747265 2.9521437804 0.1611201738

H -3.7384901943 1.7597096085 -0.5426849749

H 0.714273054 -1.0412538323 2.7699879648

H 1.6132839156 -1.9860174742 -1.686582181

H -0.812112494 -1.7918497582 -1.8537085452

H -0.6898403611 -0.3428579456 -2.1230246543

H -0.0760301081 -1.8892307026 -3.4238497567

H 3.0809399974 -0.1226821398 -2.4471324355

H 1.6726124424 0.7530994635 -3.0570430075

H 2.1492277835 -0.7775566508 -3.7904426355

H 3.7789173844 -1.2999721695 0.8587443371

Conformation no. 2

C -0.6457129091 -0.4593212412 1.0649760569

C -0.7533821425 -1.0554243189 -0.3683603251

O 0.2455271287 -0.3959077841 -1.1311468808

C 1.1992680892 0.2574045314 -0.3035521268

C 0.406576326 0.6508190248 0.9582518184

N -1.9872816528 0.0004875121 1.3606913285

C -2.8612907423 -0.0976148793 0.3336879415

C -2.1557612951 -0.7174293564 -0.8533203998

O -4.0320752987 0.2550206037 0.3609912729

C -2.32926275 0.5389972351 2.6592895932

O -0.5730231598 -2.4388912379 -0.4218284538

C 1.8321789388 1.4179343576 -1.0958129971

C 2.6181126183 0.8936210096 -2.3008830844

C 0.7977602819 2.4446240974 -1.554271274

C 2.2680581388 -0.7580292093 0.1292446676

O 2.1439704184 -1.9583112514 0.0524661829

O 3.338074168 -0.1808133643 0.6656307706

H -0.3503012253 -1.2328153043 1.7769402354

H -0.0861949116 1.6072831519 0.7922131224

H 1.0386554075 0.7386828577 1.8406900809

H -2.6913895942 -1.6075012976 -1.1809705301

H -2.1282407235 -0.0048239763 -1.6774346034

H -3.3127978433 0.7965247413 2.6578720491

H -1.743940143 1.4359430324 2.8745127985

H -2.1386660739 -0.2018439154 3.4390269015

H 0.368853583 -2.6041265085 -0.2445170521

H 2.5305479353 1.907239258 -0.412272086

H 3.1041123218 1.7228613271 -2.8176383236

H 3.3987367049 0.1860265344 -2.0133312281

H 1.9523844133 0.397994713 -3.0100918686

H 0.0427988087 1.9800081218 -2.190843661

H 1.2969642334 3.221446933 -2.1363514362

H 0.2923275121 2.939222262 -0.7244679462

H 3.9508719263 -0.8690575235 0.969116017

Conformation no. 3

C -0.7473444626 -0.5121023334 1.2730952189

C -0.5142641277 -1.3690948027 -0.0443006501

O 0.2371231633 -0.4818158942 -0.8509665442

C 1.0236380902 0.4184011121 -0.0801305116

C 0.3663574037 0.4603005269 1.3286599817

N -2.0812687136 -0.0378695448 1.0943765425

C -2.7723862148 -0.562656943 0.0127939201

C -1.8883941168 -1.5658119871 -0.6525599806

O -3.9259375126 -0.2894882534 -0.2467143643

C -2.6952552379 0.8143492944 2.091791206

O 0.1497248495 -2.5888091063 0.107434074

C 1.088375104 1.7680129654 -0.8209702142

C 1.8110340184 1.6328911529 -2.1632314818

C -0.3000969425 2.3684242986 -1.0336093314

C 2.4172466251 -0.1903538414 0.1089149926

O 2.6691255157 -1.3664811741 -0.0165640694

O 3.3195012417 0.6997771605 0.5057852707

H -0.7264014291 -1.2561675599 2.137012911

H -0.0183899663 1.4533721297 1.5481519128

H 1.084162479 0.2112848101 2.111909497

H -2.2595509306 -2.5697538454 -0.439364633

H -1.8874511632 -1.4155218148 -1.7308047249

H -2.1559893494 1.7579730012 2.1910515741

H -2.7098403303 0.3141633886 3.0636614331

H -3.7151250448 1.0249675002 1.7808292526

H 1.1055608134 -2.4046307443 0.0921234916

H 1.6664363983 2.438638356 -0.1797653406

H 2.8301449827 1.128039338 -2.0500453844

H 1.2681094684 0.9571438634 -2.8270193984

H 1.8726100643 2.6066863391 -2.6522319975

H -0.2032352098 3.3462863012 -1.5088000978

H -0.8485376414 2.5118541376 -0.1018848898

H -0.9128955846 1.7342821679 -1.6877159567

H 4.1596192826 0.2457090068 0.6123086922

Conformation no. 4

C -0.8512321536 1.0712248334 -0.0016323068

C -0.1076676402 0.5081024942 1.2201167246

O 0.439029779 -0.7171634579 0.7841383899

C 0.8304052017 -0.5975247183 -0.5850825268

C -0.2050693789 0.3660297728 -1.2024049425

N -2.2331289869 0.6933920156 0.2565684414

C -2.4688093959 0.2407467808 1.5101293039

C -1.1608459637 0.2245525344 2.2759697531

O -3.5601987303 -0.0786721136 1.9590352163

C -3.289680145 0.9894879939 -0.6872848965

O 0.8998431165 1.412537419 1.5826482351

C 0.7849976476 -2.0115489123 -1.2113712829

C 1.1224523026 -2.0004056242 -2.7020121715

C 1.6123046435 -3.0339304517 -0.4660428984

C 2.2477063684 -0.0173502033 -0.6891541943

O 2.6339425389 0.6494991255 -1.6163219079

O 3.0409627127 -0.3742274139 0.3227448204

H -0.7583894324 2.1569895581 -0.0567112855

H -0.9516484208 -0.2042172153 -1.7535236573

H 0.2615176481 1.0658633602 -1.8904496582

H -1.1874011719 1.0180316075 3.0246340436

H -1.0080499253 -0.7294127141 2.7781964659

H -3.1206562035 0.477771244 -1.6360181498

H -3.3464393723 2.0654105864 -0.8728882479

H -4.2317619501 0.6483675802 -0.2647172409

H 1.471467422 0.9904338509 2.1256410451

H -0.2635837186 -2.3048350475 -1.0981664083

H 0.5054324128 -1.2964361457 -3.2627815491

H 2.1695648442 -1.7411726473 -2.8731129212

H 0.9554898369 -2.995313537 -3.1185107856

H 1.4220308649 -3.0384788721 0.601477873

H 2.70760398 -2.837199975 -0.5953953827

H 1.4376388711 -4.0315660996 -0.8606552864

H 3.9217023184 -0.0027932938 0.160271188

Conformation no. 5

C -0.8571294673 -0.5125795704 0.8592864717

C -1.173295553 -0.5824356783 -0.6627626545

O -0.0386146734 -0.0218686928 -1.3050123325

C 1.0900803999 0.0362904128 -0.4337962107

C 0.4691068639 0.2327363034 0.960018518

N -1.9971286729 0.1521194777 1.4368655808

C -2.8849301576 0.6352388537 0.5371201142

C -2.4183035367 0.2718041372 -0.8554355223

O -3.899616907 1.2623458821 0.8077749908

C -2.1342051453 0.3149012904 2.868054966

O -1.4243573266 -1.8683860094 -1.1462147334

C 1.9629442911 1.2115121248 -0.9200882301

C 3.0328136844 1.6760684349 0.0681349149

C 2.5612616894 0.935756085 -2.2988270131

C 1.8022224833 -1.324288268 -0.471214994

O 1.3238226201 -2.330867108 -0.9420298623

O 2.9921726051 -1.309825443 0.1164930511

H -0.7772955693 -1.5377159056 1.2688822559

H 0.2738220632 1.2975669144 1.1017540852

H 1.1105812331 -0.1127426123 1.7688675911

H -3.2013090699 -0.2737274804 -1.3804745576

H -2.1218184614 1.1796157406 -1.4143047888

H -3.0797841255 0.8130871598 3.0686126523

H -1.3196054052 0.9211632874 3.2712929585

H -2.126535612 -0.659257906 3.3618459621

H -0.5700056754 -2.3326763195 -1.158818123

H 1.2352435094 2.0221711397 -1.0236000106

H 2.6199812607 1.8791292934 1.0576863848

H 3.8325659714 0.9443875395 0.1751326911

H 3.122509231 2.6048218603 -0.3010375301

H 1.7972975467 0.6336513981 -3.1249047441

H 3.3225942254 0.150874602 -2.251618364

H 3.0469445991 1.8361001896 -2.6812453293

H 3.3639211611 -2.2060719802 0.0974224618

Conformation no. 6

C -0.6792918131 1.1912052668 0.2666768802

C -0.1766997902 0.2943426988 1.4180315218

O 0.4714007205 -0.7883102356 0.7620660493

C 0.9918337212 -0.4119847772 -0.5233655898

C 0.198971782 0.849168875 -0.939663972

N -2.0758848123 0.8021930853 0.1300001787

C -2.5440164419 0.0321176452 1.1403810127

C -1.4165481537 -0.2277448438 2.1148204521

O -3.6945755266 -0.1243611756 1.2580308112

C -2.934806443 1.3877950892 -0.876654104

O 0.6679866659 0.931098263 2.3250193197

C 0.8488774727 -1.6125922033 -1.4741454755

C -0.5796274191 -2.15498758 -1.4882130363

C 1.3107731221 -1.2807813138 -2.8956775029

C 2.4563484835 -0.0292836681 -0.2261259888

O 2.7470819854 0.9503668476 0.4245631605

O 3.3675625801 -0.8814882933 -0.6701225669

H -0.6125019054 2.2517608604 0.5322623546

H -0.4071263219 0.6490066843 -1.8195655303

H 0.8639872025 1.6745181288 -1.1829612055

H -1.6088592184 0.3328507585 3.0309586257

H -1.127321858 -1.2852041913 2.3611155307

H -2.6015412213 1.1263467552 -1.8823499911

H -2.94567041 2.4768192404 -0.7819914961

H -3.941912391 1.0047019787 -0.7300001867

H 1.4656345889 1.2120451156 1.8470634441

H 1.4986637904 -2.3972485224 -1.0790783587

H -0.9058487066 -2.451265999 -0.4928586629

H -1.291825879 -1.4216903982 -1.8755068765

H -0.6271604105 -3.0300146371 -2.1393731202

H 0.6470915916 -0.5555071125 -3.3728245776

H 1.2912725594 -2.1867784782 -3.504011612

H 2.3212545036 -0.8863891679 -2.9234436821

H 4.2446781102 -0.5796054883 -0.3841358314

Conformation no. 7

C -0.1939259927 0.1212651777 1.3346188896

C -0.8290865791 -1.0742809177 0.6183094482

O -0.5217778517 -0.8888379256 -0.7450795495

C 0.7750506749 -0.297217512 -0.8623305387

C 0.9079222321 0.6082485394 0.3799808842

N -1.3151205932 1.0449548766 1.4849783518

C -2.5266216118 0.4989672444 1.2261219564

C -2.3300882959 -0.9428053867 0.8017085983

O -3.6031676996 1.0667249955 1.3402046976

C -1.1490095372 2.356982143 2.0717318769

O -0.2471589419 -2.2490827441 1.1143885074

C 0.8307709376 0.4274341204 -2.2260512623

C -0.282705835 1.4616881277 -2.3783151157

C 2.2127128254 1.0554586575 -2.4851754524

C 1.7998017693 -1.4462381524 -0.9149263614

O 1.6396753737 -2.441295352 -1.5726402609

O 2.9117021312 -1.2186133672 -0.2094457648

H 0.1989676957 -0.1537519177 2.3134121273

H 0.7450974055 1.6476473222 0.1037136083

H 1.8923239413 0.5361255684 0.8313641824

H -2.6844530691 -1.5914590114 1.6045915645

H -2.8871093579 -1.1681957278 -0.1063379034

H -2.122963264 2.8386159659 2.114491786

H -0.4764478252 2.9718340222 1.471619418

H -0.7427811286 2.2751085092 3.0834162627

H -0.5753231105 -2.9977514254 0.6015012046

H 0.6643611902 -0.3557916633 -2.9716775718

H -0.1302516106 2.327211495 -1.7292675898

H -0.294426337 1.8272328076 -3.4070740295

H -1.2602385191 1.0337708858 -2.158512284

H 2.2017814391 1.5394171512 -3.4631538203

H 3.0073853815 0.3201026252 -2.4818933156

H 2.4361248401 1.8184205972 -1.7384765538

H 3.5153995717 -1.9665600951 -0.3126087204

Conformation no. 8

C -0.600229548 0.645125363 0.9724294173

C -0.6701086726 -0.9072126176 0.9469120387

O 0.0688745587 -1.3066479303 -0.1941249998

C 0.8475648061 -0.2327926789 -0.7260203411

C 0.0440242579 1.0247470979 -0.3638884138

N -1.9775123383 1.0605199833 1.1358630875

C -2.8838254648 0.0602518804 1.0472128992

C -2.1499949951 -1.2462303543 0.8314451789

O -4.096962556 0.1914143803 1.1253269233

C -2.3305405439 2.4504506138 1.3289577447

O -0.1633206417 -1.5197960328 2.0984109848

C 0.9844889273 -0.4886861231 -2.2420228369

C 1.5498191115 0.6936378794 -3.0266962582

C 1.7126745595 -1.767929192 -2.5279231806

C 2.2293161301 -0.1604370599 -0.0608947533

O 3.0601444678 0.6626261908 -0.3398738764

O 2.4283504312 -1.0908628612 0.8863379086

H -0.0030331508 0.9890686529 1.8204671126

H -0.7304359883 1.1728012119 -1.119287147

H 0.6605928517 1.9191292394 -0.3150933795

H -2.4561749982 -1.9791046558 1.5764416131

H -2.3865389256 -1.635833508 -0.1587172022

H -1.8262157921 2.8493133312 2.211692956

H -3.4070038815 2.5150185308 1.4690671345

H -2.0477121061 3.0491992675 0.4599299647

H 0.801074286 -1.4445054586 2.0742612129

H -0.050455185 -0.6454300862 -2.5614222997

H 0.9860971956 1.1216016122 -2.8516730359

H 2.5933318582 0.8813672706 -2.7732247508

H 1.4948270365 0.4706807226 -4.0941305657

H 1.358768707 -2.6210887012 -1.9880753205

H 2.8231492721 -1.658147212 -2.2498753724

H 1.7381227972 -1.993561977 -3.5954353976

Conformation no. 9

C -0.3889271009 -0.1220867117 1.2136614722

C -0.2803362673 -1.3385722855 -0.2000467843

O 0.2019030831 -0.282061431 -0.9988893636

C 1.0633727224 0.5674409125 -0.245164601

C 0.6570278181 0.3650573392 1.2377508171

N -1.7761218591 -0.3139557454 1.2712385777

C -2.5602188214 -0.8200874811 0.2128606015

C -1.6988999535 -1.6583027461 -0.6360582909

O -3.7656569123 -0.6494748059 0.1806934702

C -2.3119936064 0.3674798278 2.4300301219

O 0.6193476592 -2.4122899429 -0.1781214764

C 0.8969589279 2.0125595006 -0.7553130483

C 1.4326177817 2.2026708722 -2.1770361718

C -0.5573816288 2.4817923439 -0.6840825547

C 2.5319722704 0.1748097812 -0.4310166348

O 3.41513131 0.6147864078 0.2626552193

O 2.7545351221 -0.6565475041 -1.4487394352

H -0.1983365826 -1.5089766987 1.9771276389

H 0.2619767337 1.2831298345 1.6668082425

H 1.5168217173 0.1269989076 1.8380627083

H -1.9299393743 -2.7111708508 -0.4649949233

H -1.8857096825 -1.4199386987 -1.6818474156

H -1.8157487749 1.3264127559 2.5864888154

H -2.1844770931 -0.2439359143 3.3275989005

H -3.3722053344 0.5430268352 2.2642812187

H 0.9217683012 -2.5725053542 -1.0803421982

H 1.4975201673 2.6356739236 -0.0781224862

H 1.2821781213 3.2350688501 -2.4973117208

H 2.5006112583 1.9897262195 -2.2523212194

H 0.9046675614 1.5525053578 -2.8779367471

H -0.6192769019 3.1209288442 -0.9787579653

H -0.9812588912 2.3971105408 0.3167240075

H -1.1796663337 1.9007445268 -1.3663774351

H 3.7089835517 -0.8206741274 -1.5014824198

Conformation no. 10

C -0.426782519 0.9912402605 0.2374981535

C -0.8550518708 -0.2678788263 1.0339044765

O 0.0212233438 -1.3020947888 0.6090219436

C 1.1511278381 -0.8001313589 -0.0996684136

C 0.6471011803 0.5084314982 -0.739422774

N -1.6515128622 1.4555600585 -0.385713594

C -2.7158531481 0.6385242057 -0.2171242801

C -2.2970313048 -0.5432574603 0.6308589728

O -3.8341861815 0.8251433425 -0.6762308944

C -1.695308323 2.6877691953 -1.1425922183

O -0.7928816821 -0.1098516154 2.4182193282

C 1.6237921584 -1.8751238089 -1.1101684902

C 0.4369859242 -2.5953003254 -1.752958355

C 2.53431499 -1.3099350469 -2.2055481134

C 2.2220116754 -0.4482495126 0.9495003393

O 1.9587866477 -0.1161722733 2.0833566014

O 3.4701647126 -0.4981058428 0.503480871

H -0.0425632988 1.7611881463 0.9146314121

H 0.1881472607 0.2746018457 -1.700532918

H 1.442483266 1.2330312827 -0.9090757178

H -2.9476552886 -0.6280136263 1.500260712

H -2.3764320521 -1.4594675056 0.0460137911

H -1.3904072386 3.52827598 -0.5152045721

H -2.7153352102 2.8449330351 -1.4851418051

H -1.0320701253 2.6358739237 -2.0092384077

H 0.1501905804 -0.0191072776 2.6384512681

H 2.1939097842 -2.6162202877 -0.5410647903

H -0.2089097479 -1.9003567822 -2.296518287

H 0.8103685194 -3.3255998175 -2.473679235

H -0.1711899463 -3.1171372054 -1.0169832733

H 3.3892204441 -0.7692127477 -1.8059868876

H 1.9807887236 -0.6414038972 -2.869361764

H 2.9121118947 -2.1323486488 -2.8158345236

H 4.0680110216 -0.2216336188 1.2162959047

Conformation no. 11

C 0.0384971129 -0.612902348 1.5325112899

C 0.7013958502 -1.3939537794 0.3875792012

O 0.5009957494 -0.585292856 -0.7581536318

C 0.2803246796 0.7985952166 -0.4486422842

C 0.1155108489 0.8564915953 1.0947371685

N -1.3080721215 -1.1618355787 1.5659060651

C -1.4370985181 -2.3330516701 0.8964125328

C -0.1104190625 -2.665156793 0.2494380436

O -2.449869768 -3.0155327196 0.8461250476

C -2.3321886182 -0.6414740417 2.4467927957

O 2.0552668626 -1.6792110149 0.5717455758

C -0.9755537361 1.2435501257 -1.2256382845

C -1.4244456804 2.6816068288 -0.9559780082

C -0.8378157869 0.994400125 -2.7282092593

C 1.544399369 1.5747185266 -0.125380501

O 1.5850527801 2.6593852249 -1.3480146826

O 2.6609331383 0.9121381474 -0.4769138927

H 0.5436223871 -0.7707574893 2.4860712518

H -0.7683374414 1.4201893289 1.3813404236

H 0.9699626527 1.3345612557 1.5727167324

H 0.3565646338 -3.4808167636 0.8048290356

H -0.1210762432 -2.9698580419 -0.7884371797

H -3.1914302393 -1.3062828179 2.3985658305

H -2.6448387128 0.3592671233 2.1441679119

H -1.9649312617 -0.6011155977 3.4754406923

H 2.5596991701 -0.8784132027 0.3662129334

H -1.7550964479 0.5744474936 -0.8458133966

H -1.5097695223 2.9059023105 0.10858151

H -0.7382596457 3.4010550753 -1.4001787867

H -2.4110875234 2.8331659365 -1.3986980822

H -1.7895490034 1.1960861235 -3.2240471196

H -0.5590735045 -0.0380909904 -2.9381016797

H -0.0876836517 1.6544140087 -3.1692217915

H 3.4348787842 1.4568638185 -0.6868664615

Conformation no. 12

C -0.5466219483 0.312367413 1.1009752005

C -0.1000843038 -1.0743179975 0.8532114775

O 0.5642022836 -1.0718103911 -0.4001380074

C 0.8084148878 0.2483820158 -0.8778459292

C -0.2974949989 1.1039902661 -0.2319988769

N -1.9312022864 0.278358186 1.4895283889

C -2.4630943554 -0.9594584226 1.3412772683

C -1.3842155123 -1.8923926522 0.836750148

O -3.6211320754 -1.2713637682 1.5833390075

C -2.6917560435 1.425917454 1.9444091879

O 0.7366796609 -1.5881307584 1.8471120841

C 0.8274697087 0.2256041257 -2.4195986222

C 1.9627034686 -0.6618960832 -2.9379463602

C -0.5028418311 -0.2249866967 -3.0193061691

C 2.1593628031 0.7908821536 -0.3839122731

O 2.6614045202 1.8009467965 -0.7995514629

O 2.7148461207 0.0600557735 0.5949473529

H 0.0330728441 0.8368964389 1.9131240125

H -1.19786124 1.0570255222 -0.8423924713

H -0.0067875545 2.147120088 -0.1258514847

H -1.3122748629 -2.7727778754 1.4734972136

H -1.6325263304 -2.2135463596 -0.1749240831

H -2.7597764218 2.1834141312 1.1601162528

H -2.2156712036 1.8684347376 2.8220673113

H -3.6941248627 1.0955036428 2.2066350358

H 1.5838827182 -1.1246195534 1.7831207875

H 1.0238320565 1.2552797401 -2.729516435

H 2.001806113 -0.127257722 -4.0276060409

H 2.9395209187 -0.3487152949 -2.5632115182

H 1.802882649 -1.7022477693 -2.6481332455

H -1.323116041 0.4582383112 -2.7984514683

H -0.7750602466 -1.2201031231 -2.6622810489

H -0.128690091 -0.273646489 -4.1056873156

H 3.5375206266 0.491653748 0.8712420486

Conformation no. 13

C 0.9614440596 0.1212767009 -0.9827202415

C 1.0691450904 -0.7954969811 0.248644671

O -0.0839278402 -0.5289910213 0.9955096601

C -1.1807166098 -0.2621956112 0.1164215016

C -0.5414007697 0.384277026 -1.1319492302

N 1.7499129033 1.2849821241 -0.5824357825

C 2.5261084039 1.0833172929 0.508912792

C 2.2832135534 -0.3203825792 1.0251412105

O 3.3111817752 1.8126896364 0.9827743506

C 1.8655533048 2.4560601283 -1.4251000732

O 1.0434928123 -2.1645165031 -0.0809839703

C -2.1521344769 0.6930604112 0.8495476409

C -3.3728965845 1.0369638506 -0.0039480884

C -2.5712819825 0.2037942873 2.2340053609

C -1.892856121 -1.5728963362 -0.2122416496

O -2.3764875192 -1.7980470028 -1.3329284195

O -1.9822451129 -2.425177842 0.7692441069

H 1.3930092063 -0.3274797987 -1.878517123

H -0.7493009676 1.4528531452 -1.1473321888

H -0.9383850691 -0.1276936888 -2.0466054561

H 3.1628154006 -0.9286635764 0.802829923

H 2.1186146275 -0.3309603558 2.1011320442

H 0.8978600042 2.942515441 -1.5559388107

H 2.2582390849 2.1824356889 -2.4080360356

H 2.5491912867 3.1538898875 -0.9475987104

H 1.7797303574 -2.374495439 -0.6661126988

H -1.5602822522 1.6040383001 0.9854749576

H -4.0191511374 0.1674002078 -0.1465227109

H -3.9623688368 1.8061389657 0.4977893935

H -3.0986273097 1.4170078036 -0.9896342586

H -3.1194286038 1.0010112285 2.7441590068

H -1.7117996919 -0.0633980571 2.8438147029

H -3.2324442525 -0.6647095642 2.1735652169

H -2.4672095857 -3.2083134728 0.4670576189

Conformation no. 14

C 0.9710194146 -0.312077438 -0.5197557145

C 0.4860932543 -1.1510520198 0.726686923

O -0.8760261144 -0.7739741647 0.9228922264

C -1.3624533315 0.0619493022 -0.1436825919

C -0.1150160433 0.6757848283 -0.7798764212

N 2.2621282597 0.1480895271 -0.123380289

C 2.545955003 0.0063399273 1.1929212856

C 1.3850455744 -0.6973004551 1.8676543678

O 3.5627409674 0.3127608948 1.7466355663

C 3.1344863772 0.8099993719 -1.0697668559

O 0.6019704188 -2.5371550918 0.613776423

C -2.3771879053 1.069276571 0.4539028071

C -1.8117852396 1.7910811428 1.6761240409

C -2.8734181267 2.0732898259 -0.5877441723

C -2.1112303556 -0.8497121556 -1.1331213877

O -2.3296817979 -0.5663872612 -2.2807318698

O -2.5455546315 -1.9974031863 -0.6078215036

H 1.0859837933 -1.0435510999 -1.3754541256

H 0.1414984679 1.6063594946 -0.2736509045

H -0.2672996078 0.8120694873 -1.8358451984

H 1.7369813502 -1.5342257422 2.4680533464

H 0.8715243701 0.0099975315 2.5189171378

H 3.3714284866 0.1398699487 -1.8986917222

H 4.0521458719 1.0848733827 -0.5551691791

H 2.6638212132 1.7122734795 -1.4670260749

H 0.2826125262 -2.8397692833 -0.2441283596

H -3.2336401512 0.4753977486 0.7916305262

H -0.9630611192 2.428676175 1.4171021861

H -2.584912668 2.4356979282 2.0983945985

H -1.4955416993 1.0904068867 2.4473558911

H -3.6332535554 2.713966827 -0.1373247244

H -3.3136333193 1.5874862389 -1.4578881661

H -2.0612471901 2.7178066799 -0.9322074717

H -2.2142186027 -2.0587453018 0.1265464961

Conformation no. 15

C -1.0019042656 -0.1277857267 0.8797230296

C -0.7451336345 -1.1871253117 -0.4121074654

O 0.3665948814 -0.5594231289 -1.0047469968

C 1.2411948005 -0.0153112499 0.0015611388

C 0.3623222226 0.2019680743 1.2479101434

N -2.0127023159 0.5809477959 0.4572112963

C -2.5978210685 0.2887309617 -0.1260208324

C -1.9622423471 -0.9663151592 -1.289958826

O -3.5101662129 0.9180084395 -1.2391012585

C -2.4968607639 1.6077824852 1.350861125

O -0.3724221387 -2.5222460104 -0.1851411459

C 1.8872867997 1.2128229374 -0.6005345976

C 0.8280462247 2.2550055303 -1.0680242024

C 2.8770326442 1.9258524514 0.3539749969

C 2.3480162247 -1.0501921775 0.2656116015

O 3.001930742 -1.0869264219 1.2744685335

O 2.5853431261 -1.8877495793 -0.7429322825

H -1.4015666764 -1.0188768813 1.6771293622

H 0.2839507479 1.2569157899 1.5008819584

H 0.7896551037 -0.3052332892 2.1097105464

H -2.6761487019 -1.7876259375 -1.1964771684

H -1.703211981 -0.8514398108 -2.3409006694

H -1.7052893713 2.3086121915 1.6201585758

H -2.8964256908 1.1598613821 2.2644695428

H -3.2887711812 2.1516899551 0.8416246392

H -1.0936593743 -3.0031199278 0.2361893054

H 2.4391141525 0.9229463381 -1.485534977

H 0.2695731912 2.6757120479 -0.2285392274

H 1.3175651237 3.0846265573 -1.5813138123

H 0.119885164 1.7984133645 -1.7580353261

H 3.3117937224 2.8002126622 -0.1333522379

H 3.6914099857 1.2628917789 0.6440660485

H 2.1206025953 2.2701558478 1.2649116673

H 1.8701578924 -1.7888901739 -1.3956161302

Conformation no. 16

C 0.8430748936 -0.4120933973 -0.8905915316

C 0.6232312648 -1.0936876748 0.4872942667

O -0.6081030777 -0.5733318395 0.9867192503

C -1.1969851298 0.401935412 0.1016158416

C -0.0903489962 0.7804030765 -0.8895422764

N 2.2581278774 -0.1300610993 -0.9038848612

C 2.8624625976 -0.2381456524 0.3127558274

C 1.8296494532 -0.6834933279 1.3208031584

O 4.0373221451 0.0006679935 0.5356692081

C 2.9304666102 0.3230243505 -2.1033623805

O 0.5840313584 -2.4858421484 0.4578031084

C -1.6835024607 1.5906312693 0.9625234064

C -2.1937212772 2.7641271969 0.127511907

C -2.7121038768 1.1686758599 2.0024215371

C -2.3917158656 -0.2660739795 -0.5995801616

O -2.9431294404 0.2010276983 -1.5606347943

O -2.8073401294 -1.4041498117 -0.0439698299

H 0.599503065 -1.1251823256 -1.7000127755

H 0.4463704121 1.6533386576 -0.5137371285

H -0.4912331465 1.0201169677 -1.8716383505

H 2.2073972598 -1.5030962062 1.9291632074

H 1.5916641057 0.1571565037 1.9734143567

H 2.8030849664 -0.4064670124 -2.9056109132

H 3.9894401259 0.4329064625 -1.881708996

H 2.5331280309 1.2859996605 -2.4335459318

H 0.0289004985 -2.7977058936 -0.2125053701

H -0.7814726245 1.9109651739 1.4936379232

H -1.4558176842 3.1081178497 -0.5989408595

H -3.1044534809 2.5019298152 -0.4128328121

H -2.423058058 3.601748075 0.788734777

H -2.368351351 0.3340651259 2.6094549069

H -3.6664663372 0.8816190881 1.5330786846

H -2.9125038075 2.0049977389 2.6712933711

H -2.2065171611 -1.6137301769 0.1246552295

Conformation no. 17

C 1.0441450376 -0.2126737414 -0.8178200168

C 0.5273035398 -1.3194866368 0.182661898

O -0.6581099021 -0.7422330643 0.7239840607

C -1.2715934819 0.1903875912 -0.1820952727

C -0.184369891 0.5630026283 -1.2092866763

N 2.0441282112 0.4529811447 -0.040100081

C 2.393541003 -0.1520158743 1.1211124966

C 1.563823909 -1.4076478435 1.2838598116

O 3.2536832261 0.2122804714 1.8949647713

C 2.7722153114 1.5748279988 -0.5932799838

O 0.2781896308 -2.5794044274 -0.3624346406

C -1.8261357578 1.362537808 0.668357127

C -0.7306809618 2.0051247927 1.5173580457

C -2.5476612017 2.4181182001 -0.1699884399

C -2.4542859487 -0.531251807 -0.8511219509

O -2.9668469044 -0.1707621495 -1.8774446775

O -2.9073579179 -1.5936034424 -0.1853683561

H 1.5145161986 -0.7319220432 -1.6867071222

H 0.0324824362 1.628567054 -1.1901307271

H -0.5113171584 0.3212251542 -2.2195035264

H 2.209281098 -2.2748564975 1.1387119991

H 1.1155494733 -1.4635742654 2.2741155947

H 3.2890453832 1.2803514141 -1.5103988514

H 3.50354343 1.9012424328 0.1422065887

H 2.1037740003 2.4071493286 -0.8184471746

H -0.1055038472 -2.5123457995 -1.2442671248

H -2.5540846569 0.9144472703 1.3534043616

H -1.1777801495 2.7124781637 2.1706952808

H -0.2216216666 1.2729969266 2.1426181511

H 0.0150895641 2.5122479081 0.9005526934

H -2.9304779336 3.1986141835 0.4897214016

H -3.3874857475 2.0075608298 -0.7288810184

H -1.8683126658 2.8945070822 -0.8815479961

H -2.3001679597 -1.7667707969 0.5512953648
